# Quantum–Classical Correspondence Principle for Heat Distribution in Quantum Brownian Motion

**DOI:** 10.3390/e23121602

**Published:** 2021-11-26

**Authors:** Jin-Fu Chen, Tian Qiu, Hai-Tao Quan

**Affiliations:** 1 School of Physics, Peking University, Beijing 100871, China; chenjinfu@pku.edu.cn (J.-F.C.); tianqiu2016@pku.edu.cn (T.Q.); 2 Collaborative Innovation Center of Quantum Matter, Beijing 100871, China; 3 Frontiers Science Center for Nano-Optoelectronics, Peking University, Beijing 100871, China

**Keywords:** open quantum systems, phase-space formulation, quantum Brownian motion, heat statistics

## Abstract

Quantum Brownian motion, described by the Caldeira–Leggett model, brings insights to the understanding of phenomena and essence of quantum thermodynamics, especially the quantum work and heat associated with their classical counterparts. By employing the phase-space formulation approach, we study the heat distribution of a relaxation process in the quantum Brownian motion model. The analytical result of the characteristic function of heat is obtained at any relaxation time with an arbitrary friction coefficient. By taking the classical limit, such a result approaches the heat distribution of the classical Brownian motion described by the Langevin equation, indicating the quantum–classical correspondence principle for heat distribution. We also demonstrate that the fluctuating heat at any relaxation time satisfies the exchange fluctuation theorem of heat and its long-time limit reflects the complete thermalization of the system. Our research study justifies the definition of the quantum fluctuating heat via two-point measurements.

## 1. Introduction

In the past few decades, the discovery of fluctuation theorems [1,2,3,4] and the establishment of the framework of stochastic thermodynamics [5,6,7] deepened our understanding of the fluctuating nature of thermodynamic quantities (such as work, heat and entropy production) in microscopic systems [8,9,10,11,12,13]. Among various fluctuation theorems, the non-equilibrium work relation [2] sharpens our understanding of the second law of thermodynamics by presenting an elegant and precise equality associating the free energy change with the fluctuating work. Such a relation was later extended to the quantum realm based on the two-point measurement definition of the quantum fluctuating work [14,15], soon after its discovery in the classical regime. The work statistics has been widely studied in various microscopic classical and quantum systems [16,17,18,19,20,21,22,23,24,25,26]. Historically, the quantum–classical correspondence principle played an essential role in the development of the theory of quantum mechanics and the interpretation of the transition from quantum to classical world [27,28]. In Refs. [19,22,24], it is demonstrated that the existence of the quantum–classical correspondence principle for work distribution brings justification for the definition of quantum fluctuating work via two-point measurements.

Compared to work statistics, heat statistics relevant to thermal transport associated with a nonequilibrium stationary state has been extensively studied [29,30,31,32,33,34,35,36,37,38], but the heat statistics in a finite-time quantum thermodynamic process [39,40,41] and its quantum–classical correspondence have been less explored. A challenge is that the precise description of the bath dynamics requires handling a huge number of degrees of freedom of the heat bath. Different approaches have been proposed to calculate the quantum fluctuating heat and its statistics, such as the non-equilibrium Green’s function approach to quantum thermal transport [29,32,36,42,43,44] and the path-integral approach to quantum thermodynamics [45,46,47,48,49]. However, very few analytical results about the heat statistics have been obtained for the relaxation processes in open quantum systems. These analytical results are limited to either the relaxation dynamics described by the Lindblad master equation [39,40] or the long-time limit independent of the relaxation dynamics [50]. On the other hand, some results about the heat statistics in the classical Brownian motion model have been reported [51,52,53,54,55,56,57,58,59]. How the quantum and the classical heat statistics (especially associated with the relaxation dynamics in finite time) are related to each other has not been explored so far, probably due to the difficulty in studying the heat statistics in open quantum systems [60,61,62].

In this article, we study the heat statistics of a quantum Brownian motion model described by the Caldeira–Leggett Hamiltonian [48,63,64,65,66,67,68], where the heat bath is modeled as a collection of harmonic oscillators. Although it is well known that the dynamics of such an open quantum system can approach that of the classical Brownian motion in the classical limit ℏ→0 [64], less is known about the heat statistics of this model during the finite-time relaxation process. Here, we focus on the relaxation process without external driving (the Hamiltonian of the system is time-independent); thus, the quantum fluctuating heat can be defined as the difference of the system energy between the initial and the final measurements [69].Under the Ohmic spectral density, the dynamics of the composite system is exactly solvable in the continuum limit of the bath oscillators [70]. By employing the phase-space formulation approach [71,72,73], we obtain analytical results of the characteristic function of heat for the Caldeira–Leggett model at any relaxation time τ with an arbitrary friction coefficient κ . Previously, such an approach was employed to study the quantum corrections to work [74,75,76] and entropy [77,78]. Analytical results of the heat statistics bring important insights to understand the fluctuating property of heat. By taking the classical limit ℏ→0 , the heat statistics of the Caldeira–Leggett model approaches that of the classical Brownian motion model. Thus, our results verify the quantum–classical correspondence principle for heat distribution, and provide justification for the definition of the quantum fluctuating heat via two-point measurements. We also verify, from the analytical results, that the heat statistics satisfies the exchange fluctuation theorem of heat [4].

The rest of this article is organized as follows. In Section 2, we introduce the Caldeira–Leggett model and define the quantum fluctuating heat. In Section 3, the analytical results of the characteristic function of heat are obtained by employing the phase-space formulation approach. We show the quantum–classical correspondence of the heat distribution and discuss the heat distribution in the long-time limit or with the extremely weak or strong coupling strength. The conclusion is given in Section 4.

## 2. The Caldeira–Leggett Model and the Heat Statistics

### 2.1. The Caldeira–Leggett Model

The quantum Brownian motion is generally described by the Caldeira–Leggett model [64,65], where the system is modeled as a single particle moving in a specific potential and the heat bath is a collection of harmonic oscillators. For simplicity, we choose the harmonic potential for the system [66,79,80,81], where the dynamics of such an open quantum system can be solved analytically. The system relaxes to the equilibrium state at the temperature of the heat bath. We study the heat distribution of such a quantum relaxation process and analytically obtain the characteristic function of heat and its classical correspondence based on the phase-space formulation of quantum mechanics.

The total Hamiltonian of the composite system is $H_{\mathrm{tot}}=H_{S}+H_{B}+H_{SB}$ with each term being

(1) $$\begin{array}{cc} H_{S} & =\frac{1}{2}\frac{{\hat{p}}_{0}^{2}}{m_{0}}+\frac{1}{2}m_{0}\omega_{0}^{2}{\hat{q}}_{0}^{2} \end{array}$$

(2) $$\begin{array}{cc} H_{B} & =\sum_{n=1}^{N}\left(\frac{1}{2}\frac{{\hat{p}}_{n}^{2}}{m_{n}}+\frac{1}{2}m_{n}\omega_{n}^{2}{\hat{q}}_{n}^{2}\right) \end{array}$$

(3) $$\begin{array}{cc} H_{SB} & =-{\hat{q}}_{0}\sum_{n=1}^{N}\left(C_{n}{\hat{q}}_{n}\right)+\sum_{n=1}^{N}\left(\frac{C_{n}^{2}}{2m_{n}\omega_{n}^{2}}{\hat{q}}_{0}^{2}\right), \end{array}$$

where $m_{0}$ , $\omega_{0}$ , ${\hat{q}}_{0}$ and ${\hat{p}}_{0}$ ( $m_{n}$ , $\omega_{n}$ , ${\hat{q}}_{n}$ and ${\hat{p}}_{n}$ with n=1,2,3,...,N ) are the mass, frequency, position and momentum of the system (the *n*-th bath harmonic oscillator) and $C_{n}$ is the coupling strength between the system and the *n*-th bath harmonic oscillator. The counter-term $\sum_{n}\left[C_{n}^{2}/\left(2m_{n}\omega_{n}^{2}\right)\right]{\hat{q}}_{0}^{2}$ is included in the interaction Hamiltonian $H_{SB}$ to cancel the frequency shift of the system.

The spectral density is defined as $J\left(\omega \right):=\sum_{n}\left[C_{n}^{2}/\left(2m_{n}\omega_{n}\right)\right]\delta \left(\omega -\omega_{n}\right)$ . We adopt an Ohmic spectral density with the Lorentz–Drude cutoff [67]

(4) $$J\left(\omega \right)=\frac{m_{0}\kappa }{\pi }\omega \frac{\Omega_{0}^{2}}{\Omega_{0}^{2}+\omega^{2}},$$

where κ is the friction coefficient. A sufficiently large cutoff frequency $\Omega_{0}$ ( $\Omega_{0}\gg \omega_{0}$ ) is applied to ensure a finite counter-term and the dynamics with the timescale exceeding $1/\Omega_{0}$ is Markovian. Under such a spectral density, the dissipation dynamics of the Caldeira–Leggett model with a weak coupling strength $\kappa \ll \omega_{0}$ reproduces that of the classical underdamped Brownian motion when taking the classical limit ℏ→0 [64].

We assume the initial state to be a product state of the system and the heat bath

(5) $$\rho \left(0\right)=\rho_{S}\left(0\right)⊗\rho_{B}^{G},$$

which makes it possible to define the quantum fluctuating heat via two-point measurements. Here, $\rho_{S}\left(0\right)$ is the initial state of the system and $\rho_{B}^{G}=\exp\left(-\beta H_{B}\right)/Z_{B}\left(\beta \right)$ is the Gibbs distribution of the heat bath with the inverse temperature β and the partition function $Z_{B}\left(\beta \right)=\mathrm{Tr}\left[\exp\left(-\beta H_{B}\right)\right]$ .

### 2.2. The Quantum Fluctuating Heat in the Relaxation Process

We study the heat distribution of the relaxation process based on the two-point measurement definition of the quantum fluctuating heat. When no external driving is applied to the system, the Hamiltonian of the system is time-independent. Since no work is performed during the relaxation process, the quantum fluctuating heat can be defined as

(6) $$Q_{l^{\prime }l}=E_{l^{\prime }}^{S}-E_{l}^{S},$$

where $E_{l}^{S}$ ( $E_{l^{\prime }}^{S}$ ) is the eigenenergy of the system corresponding to the outcome *l* ( $l^{\prime }$ ) at the initial (final) time t=0 ( t=τ ). The two-point measurements over the heat bath can be hardly realized due to a huge number of degrees of freedom of the heat bath [20], while the measurements over the small quantum system are much easier in principle. The positive sign corresponds to the energy flowing from the heat bath to the system.

For the system prepared in an equilibrium state, no coherence exists in the initial state and the initial density matrix of the system commutes with the Hamiltonian of the system, $[\rho \left(0\right),H_{S}]=0$ . The probability of observing the transition from *l* and $l^{\prime }$ is

(7) $$p_{\tau ,l^{\prime }l}=\gamma_{\tau ,l^{\prime }l}p_{l},$$

with the conditional transition probability $\gamma_{\tau ,l^{\prime }l}=\mathrm{Tr}\left[\left({\hat{P}}_{l^{\prime }}^{S}⊗I_{B}\right)U_{\mathrm{tot}}\left(\tau \right)\left({\hat{P}}_{l}^{S}⊗\rho_{B}^{G}\right)U_{\mathrm{tot}}^{†}\left(\tau \right)\right]$ and the initial probability $p_{l}=\mathrm{Tr}\left[\rho \left(0\right){\hat{P}}_{l}^{S}\right]$ . Here, ${\hat{P}}_{l}^{S}=|l\rangle \langle l|$ is the projection operator corresponding to the outcome *l*. The heat distribution is defined as

(8) $$P_{\tau }\left(q\right):=\sum_{l^{\prime },l}\delta \left(q-Q_{l^{\prime }l}\right)p_{\tau ,l^{\prime }l}.$$

The characteristic function of heat $\chi_{\tau }\left(\nu \right)$ is defined as the Fourier transform of the heat distribution $\chi_{\tau }\left(\nu \right):=\sum_{l^{\prime },l}\exp\left[i\nu \left(E_{l^{\prime }}^{S}-E_{l}^{S}\right)\right]p_{\tau ,l^{\prime }l}$ , which can be rewritten explicitly as

(9) $$\chi {}_{\tau }\left(\nu \right)=\mathrm{Tr}\left[e^{i\nu H_{S}}U_{\mathrm{tot}}\left(\tau \right)\left(e^{-i\nu H_{S}}\rho \left(0\right)\right)U_{\mathrm{tot}}^{†}\left(\tau \right)\right],$$

where $U_{\mathrm{tot}}\left(\tau \right)=\exp\left(-iH_{\mathrm{tot}}\tau /\hbar \right)$ is the unitary time-evolution operator of the composite system.

Our goal is to analytically calculate the characteristic function $\chi {}_{\tau }\left(\nu \right)$ . Previously, the quantum–classical correspondence principle for heat statistics has been analyzed with the path-integral approach to quantum thermodynamics [48], yet the explicit result of the characteristic function (or generating function) of heat has not been obtained so far. We employ the phase-space formulation approach to solve this problem and rewrite the characteristic function Equation (9) into

(10) $$\chi {}_{\tau }\left(\nu \right)=\mathrm{Tr}\left[e^{i\nu H_{S}^{H}\left(\tau \right)}\eta \left(0\right)\right],$$

where the system Hamiltonian in the Heisenberg picture is

(11) $$H_{S}^{H}\left(\tau \right)=U_{\mathrm{tot}}^{†}\left(\tau \right)H_{S}U_{\mathrm{tot}}\left(\tau \right),$$

and the density matrix-like operator η(0) is

(12) $$\eta \left(0\right)=\left[e^{-i\nu H_{S}}\rho_{S}\left(0\right)\right]⊗\rho_{B}^{G}.$$

We express Equation (10) with the phase-space formulation of quantum mechanics [71,72,73,74,75,76]:

(13) $$\chi_{\tau }\left(\nu \right)=\frac{1}{{\left(2\pi \hbar \right)}^{N+1}}\int dz{\left[e^{i\nu H_{S}^{H}\left(\tau \right)}\right]}_{w}\left(z\right)\cdot P\left(z\right),$$

where z represents a point $z=\left[q,p\right]=\left[q_{0},...,q_{N},p_{0},...,p_{N}\right]$ in the phase space of the composite system and the integral is performed over the whole phase space. The subscript “*w*” indicates the Weyl symbol of the corresponding operator and P(z) is the Weyl symbol of the operator η(0) , which is explicitly defined as [71]

(14) $$P\left(z\right):=\int dy\langle q-\frac{y}{2}|\eta \left(0\right)|q+\frac{y}{2}\rangle e^{\frac{ip\cdot y}{\hbar }}.$$

In the following, we calculate the heat statistics Equation (13) by employing the phase-space formulation approach.

## 3. Results of the Characteristic Function of Heat

We show a sketch of the derivation of the heat statistics $\chi_{\tau }\left(\nu \right)$ with the details left in Appendix A. We specifically consider the system is initially prepared at an equilibrium state $\rho_{S}\left(0\right)=\exp\left(-\beta^{\prime }H_{S}\right)/Z_{S}\left(\beta^{\prime }\right)$ with the inverse temperature $\beta^{\prime }$ and the partition function $Z_{S}\left(\beta^{\prime }\right)=1/\left[2\sinh\left(\beta^{\prime }\hbar \omega_{0}/2\right)\right]$ . The heat bath is at the inverse temperature β , which is different from $\beta^{\prime }$ . In Equation (13), the two Weyl symbols ${\left[e^{i\nu H_{S}^{H}\left(\tau \right)}\right]}_{w}\left(z\right)$ and P(z) are obtained as

(15) $${\left[e^{i\nu H_{S}^{H}\left(\tau \right)}\right]}_{w}\left(z\right)=\frac{1}{\cos\left(\frac{\nu \hbar \omega_{0}}{2}\right)}\exp\left[\frac{i}{2\hbar }z^{T}{\tilde{\Lambda }}_{\nu z}\left(\tau \right)z\right],$$

and

(16) $$P\left(z\right)=\frac{2\sinh\left(\frac{\beta^{\prime }\hbar \omega_{0}}{2}\right)}{\cosh\left[\frac{\left(\beta^{\prime }+i\nu \right)\hbar \omega_{0}}{2}\right]}\cdot \left[\prod_{n=1}^{N}2\tanh\left(\frac{\beta \hbar \omega_{n}}{2}\right)\right]\cdot \exp\left(-\frac{1}{2\hbar }z^{T}\Lambda_{\beta z}z\right),$$

where the explicit expressions of the matrices ${\tilde{\Lambda }}_{\nu z}\left(\tau \right)$ and $\Lambda_{\beta z}$ are given in Equations (A10) and (A36), respectively.

Substituting Equations (15) and (16) into Equation (13), the characteristic function of heat at any relaxation time τ with an arbitrary friction coefficient κ is finally obtained as

(17) $$\begin{array}{cc} \chi_{\tau }\left(\nu \right)= & \left\{{\left[\left(1+i\Xi \right)\left(1-i\Theta \Xi \right)-i\Xi \left(1-\Theta -i\Theta \Xi \right)\frac{\kappa^{2}\cos\left(2{\hat{\omega }}_{0}\tau \right)-4\omega_{0}^{2}}{\left(\kappa^{2}-4\omega_{0}^{2}\right)e^{\kappa \tau }}\right]}^{2}\right.\\ \; & {\left.+\Xi^{2}{\left(1-\Theta -i\Theta \Xi \right)}^{2}\left[{\left(\frac{\kappa^{2}\cos\left(2{\hat{\omega }}_{0}\tau \right)-4\omega_{0}^{2}}{\left(\kappa^{2}-4\omega_{0}^{2}\right)e^{\kappa \tau }}\right)}^{2}-e^{-2\kappa \tau }\right]\right\}}^{\frac{1}{2}}, \end{array}$$

where the quantities Ξ and Θ are

(18) $$\begin{array}{cc} \Xi & =\frac{\tan\left(\frac{\nu \hbar \omega_{0}}{2}\right)}{\tanh\left[\frac{\left(\beta^{\prime }+i\nu \right)\hbar \omega_{0}}{2}\right]-i\tan\left(\frac{\nu \hbar \omega_{0}}{2}\right)}, \end{array}$$

(19) $$\begin{array}{cc} \Theta & =\frac{\tanh\left[\frac{\left(\beta^{\prime }+i\nu \right)\hbar \omega_{0}}{2}\right]-i\tan\left(\frac{\nu \hbar \omega_{0}}{2}\right)}{\tanh\left(\frac{\beta \hbar \omega_{0}}{2}\right)}. \end{array}$$

Induced by the friction, the frequency of the system harmonic oscillator is shifted to ${\hat{\omega }}_{0}=\sqrt{\omega_{0}^{2}-\kappa^{2}/4}$ .

From the analytical results of the heat statistics Equation (17), the average heat $\langle Q\rangle \left(\tau \right)={\left.-i\partial_{\nu }\left[\ln\chi_{\tau }\left(\nu \right)\right]\right|}_{\nu =0}$ is immediately obtained as

(20) $$\begin{array}{cc} \langle Q\rangle \left(\tau \right) & =\frac{\omega_{0}\hbar }{2}\left[\coth\left(\frac{\beta \omega_{0}\hbar }{2}\right)-\coth\left(\frac{\beta^{\prime }\omega_{0}\hbar }{2}\right)\right]\left[1-\frac{\kappa^{2}\cos\left(2{\hat{\omega }}_{0}\tau \right)-4\omega_{0}^{2}}{\left(\kappa^{2}-4\omega_{0}^{2}\right)e^{\kappa \tau }}\right], \end{array}$$

and the variance $\mathrm{Var}\left(Q\right)\left(\tau \right)={\left.-\partial_{\nu }^{2}\left[\ln\chi_{\tau }\left(\nu \right)\right]\right|}_{\nu =0}$ is

(21) $$\mathrm{Var}\left(Q\right)\left(\tau \right)=I+\mathrm{II}\cdot e^{-\kappa \tau }+\mathrm{III}\cdot e^{-2\kappa \tau },$$

with

(22) $$\begin{array}{cc} I & =\frac{\omega_{0}^{2}\hbar^{2}\left[{\mathrm{csch}}^{2}\left(\frac{\beta \omega_{0}\hbar }{2}\right)+{\mathrm{csch}}^{2}\left(\frac{\beta^{\prime }\omega_{0}\hbar }{2}\right)\right]}{4}, \end{array}$$

(23) $$\begin{array}{cc} \mathrm{II} & =\frac{\kappa^{2}\cos\left(2{\hat{\omega }}_{0}\tau \right)-4\omega_{0}^{2}}{2{\hat{\omega }}_{0}^{2}}\cdot \frac{\omega_{0}^{2}\hbar^{2}\left[\coth^{2}\left(\frac{\beta \omega_{0}\hbar }{2}\right)+{\mathrm{csch}}^{2}\left(\frac{\beta^{\prime }\omega_{0}\hbar }{2}\right)-\coth\left(\frac{\beta \omega_{0}\hbar }{2}\right)\coth\left(\frac{\beta^{\prime }\omega_{0}\hbar }{2}\right)\right]}{4} \end{array}$$

(24) $$\begin{array}{cc} \mathrm{III} & =\frac{\kappa^{4}\cos\left(4{\hat{\omega }}_{0}\tau \right)+8\omega_{0}^{2}\kappa^{2}\left[1-2\cos\left(2{\hat{\omega }}_{0}\tau \right)\right]+16\omega_{0}^{4}}{16{\hat{\omega }}_{0}^{4}}\cdot \frac{\omega_{0}^{2}\hbar^{2}{\left[\coth\left(\frac{\beta \omega_{0}\hbar }{2}\right)-\coth\left(\frac{\beta^{\prime }\omega_{0}\hbar }{2}\right)\right]}^{2}}{4}. \end{array}$$

Similarly, one can calculate the higher cumulants from the analytical results of the heat statistics. In the following, we examine the properties of the heat statistics of the quantum Brownian motion.

### 3.1. Quantum–Classical Correspondence Principle for Heat Statics and the Exchange Fluctuation Theorem of Heat

We further take the classical limit ℏ→0 or, more rigorously, $\beta \hbar \omega_{0}\to 0$ . The two quantities approach $\Xi \to \nu /\beta^{\prime }$ and $\Theta \to \beta^{\prime }/\beta$ and the characteristic function of heat (Equation (17)) becomes

(25) $$\begin{array}{cc} \chi_{\tau }^{\mathrm{cl}}\left(\nu \right)= & \left\{{\left[\left(1+i\frac{\nu }{\beta^{\prime }}\right)\left(1-i\frac{\nu }{\beta }\right)-\frac{i\nu \left(\beta -\beta^{\prime }-i\nu \right)}{\beta \beta^{\prime }}\frac{\left(\kappa^{2}\cos\left(2{\hat{\omega }}_{0}\tau \right)-4\omega_{0}^{2}\right)}{\left(\kappa^{2}-4\omega_{0}^{2}\right)e^{\kappa \tau }}\right]}^{2}\right.\\ \; & {\left.+\nu^{2}{\left(\frac{\beta -\beta^{\prime }-i\nu }{\beta \beta^{\prime }}\right)}^{2}\left[{\left(\frac{\kappa^{2}\cos\left(2{\hat{\omega }}_{0}\tau \right)-4\omega_{0}^{2}}{\left(\kappa^{2}-4\omega_{0}^{2}\right)e^{\kappa \tau }}\right)}^{2}-e^{-2\kappa \tau }\right]\right\}}^{-\frac{1}{2}}, \end{array}$$

which is consistent with the results obtained from the classical Brownian motion described by the Kramers equation (see Ref. [58] or Appendix B). The average heat is

(26) $$\begin{array}{cc} \langle Q^{\mathrm{cl}}\rangle \left(\tau \right) & =\frac{\beta^{\prime }-\beta }{\beta \beta^{\prime }}\left[1-\frac{\kappa^{2}\cos\left(2{\hat{\omega }}_{0}\tau \right)-4\omega_{0}^{2}}{\left(\kappa^{2}-4\omega_{0}^{2}\right)e^{\kappa \tau }}\right], \end{array}$$

and the variance $\mathrm{Var}\left(Q^{\mathrm{cl}}\right)\left(\tau \right)={\left.-\partial_{\nu }^{2}\left[\ln\chi_{\tau }^{\mathrm{cl}}\left(\nu \right)\right]\right|}_{\nu =0}$ is

(27) $$\begin{array}{cc} \mathrm{Var}\left(Q^{\mathrm{cl}}\right)\left(\tau \right) & =I^{\mathrm{cl}}+{\mathrm{II}}^{\mathrm{cl}}\cdot e^{-\kappa \tau }+{\mathrm{III}}^{\mathrm{cl}}\cdot e^{-2\kappa \tau }, \end{array}$$

with

(28) $$\begin{array}{cc} I^{\mathrm{cl}} & =\frac{\beta^{2}+\beta^{\prime 2}}{\beta^{2}\beta^{\prime 2}} \end{array}$$

(29) $$\begin{array}{cc} {\mathrm{II}}^{\mathrm{cl}} & =\frac{\kappa^{2}\cos\left(2{\hat{\omega }}_{0}\tau \right)-4\omega_{0}^{2}}{2{\hat{\omega }}_{0}^{2}}\cdot \frac{\beta^{2}-\beta \beta^{\prime }+\beta^{\prime 2}}{\beta^{2}\beta^{\prime 2}} \end{array}$$

(30) $$\begin{array}{cc} {\mathrm{III}}^{\mathrm{cl}} & =\frac{\kappa^{4}\cos\left(4{\hat{\omega }}_{0}\tau \right)+8\omega_{0}^{2}\kappa^{2}\left[1-2\cos\left(2{\hat{\omega }}_{0}\tau \right)\right]+16\omega_{0}^{4}}{16{\hat{\omega }}_{0}^{4}}\cdot \frac{{\left(\beta -\beta^{\prime }\right)}^{2}}{\beta^{2}\beta^{\prime 2}}. \end{array}$$

From Equation (17) (or the classical counterpart Equation (25)), one can see the characteristic function of heat exhibits the following symmetry:

(31) $$\chi_{\tau }\left(\nu \right)=\chi_{\tau }\left[-i\left(\beta -\beta^{\prime }\right)-\nu \right],$$

which shows that the heat distribution satisfies the exchange fluctuation theorem of heat in the differential form $P_{\tau }\left(Q\right)/P_{\tau }\left(-Q\right)=\exp\left[-\left(\beta -\beta^{\prime }\right)Q\right]$ [4]. By setting ν=0 , we obtain the relation $\chi_{\tau }\left[-i\left(\beta -\beta^{\prime }\right)\right]=\chi_{\tau }\left(0\right)=1$ , which is exactly the exchange fluctuation theorem of heat in the integral form $\langle \exp[\left(\beta -\beta^{\prime }\right)Q]\rangle =1$ .

### 3.2. Long-Time Limit

In the long-time limit τ→∞ , the characteristic functions of heat (Equations (17) and (25)) become

(32) $$\begin{array}{cc} \chi_{\infty }\left(\nu \right) & =\frac{\left(1-e^{-\beta^{\prime }\omega_{0}\hbar }\right)\left(1-e^{-\beta \omega_{0}\hbar }\right)}{\left(1-e^{-\left(\beta^{\prime }+i\nu \right)\omega_{0}\hbar }\right)\left(1-e^{-\left(\beta -i\nu \right)\omega_{0}\hbar }\right)}, \end{array}$$

and

(33) $$\chi_{\infty }^{\mathrm{cl}}\left(\nu \right)=\frac{\beta^{\prime }\beta }{\left(\beta^{\prime }+i\nu \right)\left(\beta -i\nu \right)}.$$

Such results, independent of the relaxation dynamics, are in the form

(34) $$\chi_{\mathrm{th}}\left(\nu \right)=\frac{Z_{S}\left(\beta^{\prime }+i\nu \right)Z_{S}\left(\beta -i\nu \right)}{Z_{S}\left(\beta^{\prime }\right)Z_{S}\left(\beta \right)},$$

reflecting complete thermalization of the system [53]. For example, the relaxation of a harmonic oscillator governed by the quantum–optical master equation gives the identical characteristic function of heat in the long-time limit [39]. In Appendix C, we demonstrate that the characteristic function of heat for any relaxation process with complete thermalization is always in the form of Equation (34). With the simple expressions (32) and (33) of the characteristic functions, the heat distributions are obtained from the inverse Fourier transform as

(35) $$P_{\infty }\left(q\right)=\left\{\begin{array}{cc} \frac{\left(1-e^{-\beta^{\prime }\omega_{0}\hbar }\right)\left(1-e^{-\beta \omega_{0}\hbar }\right)}{1-e^{-\left(\beta^{\prime }+\beta \right)\omega_{0}\hbar }}\sum_{j=0}^{\infty }\delta \left(q-j\omega_{0}\hbar \right)e^{-\beta q} & q\geq 0\\ \frac{\left(1-e^{-\beta^{\prime }\omega_{0}\hbar }\right)\left(1-e^{-\beta \omega_{0}\hbar }\right)}{1-e^{-\left(\beta^{\prime }+\beta \right)\omega_{0}\hbar }}\sum_{j=1}^{\infty }\delta \left(q+j\omega_{0}\hbar \right)e^{\beta^{\prime }q} & q<0 \end{array}\right.,$$

and

(36) $$P_{\infty }^{\mathrm{cl}}\left(q\right)=\left\{\begin{array}{cc} \frac{\beta^{\prime }\beta }{\beta^{\prime }+\beta }e^{-\beta q} & q\geq 0\\ \frac{\beta^{\prime }\beta }{\beta^{\prime }+\beta }e^{\beta^{\prime }q} & q<0 \end{array}\right.,$$

which are exactly the same as the long-time results obtained in Ref. [39].

### 3.3. Weak/Strong Coupling Limit in Finite Time

In the weak coupling limit $\kappa \ll \omega_{0}$ , the characteristic function of heat Equation (17) becomes

(37) $$\begin{array}{cc} \chi_{\tau }^{w}\left(\nu \right) & =\frac{1}{\left(1+i\Xi \right)\left(1-i\Xi \Theta \right)\left(1-e^{-\kappa \tau }\right)+e^{-\kappa \tau }}. \end{array}$$

There is only one relaxation timescale associated to κ . Such situation corresponds to the highly underdamped regime of the classical Brownian motion and a systematic method has been proposed to study the heat distribution [56], as well as the work distribution, under an external driving [82,83].

In the strong coupling limit $\kappa \gg \omega_{0}$ , the characteristic function of heat Equation (17) becomes

(38) $$\begin{array}{cc} \chi_{\tau }^{s}\left(\nu \right)= & \frac{1}{\sqrt{\left(1+i\Xi \right)\left(1-i\Xi \Theta \right)\left(1-e^{-2\kappa \tau }\right)+e^{-2\kappa \tau }}}\\ \; & \times \frac{1}{\sqrt{\left(1+i\Xi \right)\left(1-i\Xi \Theta \right)\left(1-e^{-\frac{2\omega_{0}^{2}}{\kappa }\tau }\right)+e^{-\frac{2\omega_{0}^{2}}{\kappa }\tau }}}. \end{array}$$

The relaxation timescales of the momentum (the first factor) and the coordinate (the second factor) are separated. The long-time limits of both Equations (37) and (38) are equal to Equation (32). In classical thermodynamics, the usual overdamped approximation neglects the motion of the momentum; hence, the heat statistics derived under such an approximation is incomplete [52]. Actually, the momentum degree of freedom also contributes to the heat statistics.

### 3.4. Numerical Results

**Figure 1:**
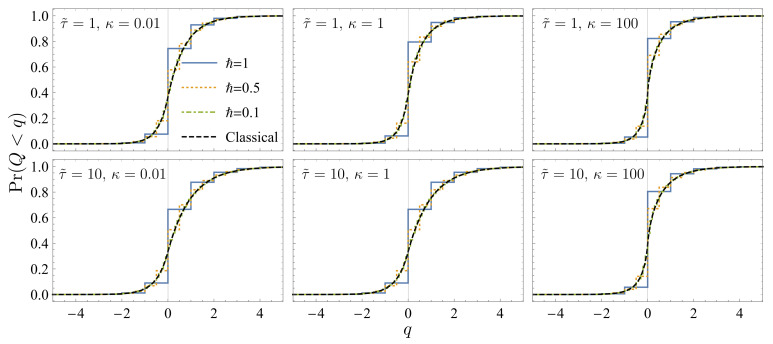
The cumulative heat distribution function Pr(Q<q) . The choices of the parameters are given in the main text. We compare the results of the Caldeira–Leggett model (blue solid, orange dotted and green dot-dashed curves) in Equation (17) and those of the classical Brownian motion (black dashed curve) in Equation (25). The rescaled relaxation time is $\tilde{\tau }=\kappa \tau =1$ in the upper subfigures and $\tilde{\tau }=10$ in the lower subfigures. The left, middle and right subfigures illustrate the results for the weak ( κ=0.01 ), intermediate ( κ=1 ) and strong coupling strength ( κ=100 ).

In Figure 1, we show the cumulative heat distribution function $\mathrm{Pr}\left(Q<q\right):=\int_{-\infty }^{q}P_{\tau }\left(q^{\prime }\right)dq^{\prime }$ with different friction coefficients κ=0.01,1 and 100, at the rescaled relaxation time $\tilde{\tau }=\kappa \tau =1$ and 10. We set the mass $m_{0}=1$ and the frequency $\omega_{0}=1$ for the system harmonic oscillator, the inverse temperatures β=1 and $\beta^{\prime }=2$ for the initial equilibrium states of the heat bath and the system, respectively. The Planck constant is set to be ℏ=1,0.5,0.1 . With the decrease in *ℏ*, the quantum result Equation (17) approaches the classical result Equation (25). Thus, the quantum–classical correspondence of the heat distribution is demonstrated for generic values of the friction coefficient κ .

For κ=0.01 and 1, complete thermalization is achieved at $\tilde{\tau }=10$ . The left-lower and middle-lower subfigures show the identical distribution characterized by Equations (35) and (36). For κ=100 , the momentum degree of freedom is thermalized $\exp(-2\tilde{\tau })\approx 0$ in Equation (38), while the coordinate degree of freedom remains frozen $\exp[-2\left(\omega_{0}^{2}/\kappa^{2}\right)\tilde{\tau }]\approx 1$ in Equation (38). Thus, the distribution in the right-lower subfigure is different from the middle-lower subfigure.

**Figure 2:**
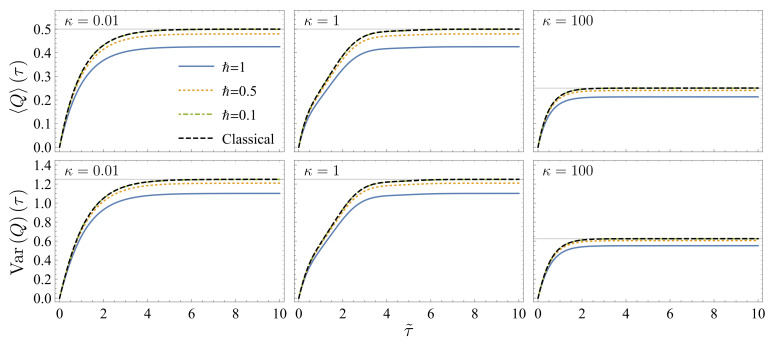
The evolution of the mean value $\langle Q\rangle \left(\tau \right)$ (**upper subfigures**) and the variance $\mathrm{Var}\left(Q\right)\left(\tau \right)$ (**lower subfigures**) of the heat statistics as functions of the rescaled time $\tilde{\tau }=\kappa \tau$ .

In Figure 2, we illustrate the results of the mean value $\langle Q\rangle \left(\tau \right)$ and the variance $\mathrm{Var}\left(Q\right)\left(\tau \right)$ with different friction coefficients κ=0.01,1 and 100. The parameters are the same as those in Figure 1. The quantum results approach the classical results with the decrease in *ℏ*. For κ=0.01 and 1 (left and middle subfigures), complete thermalization is reached when $\tilde{\tau }>5$ . The mean value and the variance approach $\lim_{\tau \to \infty }\langle Q^{\mathrm{cl}}\rangle \left(\tau \right)=1/\beta -1/\beta^{\prime }$ and $\lim_{\tau \to \infty }\mathrm{Var}\left(Q^{\mathrm{cl}}\right)\left(\tau \right)=1/\beta^{2}+1/\beta^{\prime 2}$ (gray horizontal lines). For κ=100 (right subfigures), only the momentum degree of freedom is thermalized at this timescale. Thus, the mean value and the variance take half value of their long-time limits. When the coordinate degree of freedom is also thermalized in the long-time limit ( $\tilde{\tau }\gg \kappa^{2}/\omega_{0}^{2}=10^{4}$ ), the mean value and the variance are expected to approach the same values as those in the middle subfigures.

## 4. Conclusions

Previously, the heat statistics of the relaxation processes has been studied analytically in open quantum systems described by the Lindblad master equation [39,40,50]. However, due to the rotating wave approximation and other approximations, such quantum systems do not possess a well-defined classical counterpart. Hence, the quantum–classical correspondence principle for heat distribution has not been well established.

In this paper, we study the heat statistics of the quantum Brownian motion model described by the Caldeira–Leggett Hamiltonian, in which the bath dynamics is explicitly considered. By employing the phase-space formulation approach, we obtain the analytical expressions of the characteristic function of heat at any relaxation time τ with an arbitrary friction coefficient κ . The analytical results of heat statistics bring important insights to the studies of quantum thermodynamics. For example, in the classical limit, our results approach the heat statistics of the classical Brownian motion. Thus, the quantum–classical correspondence principle for heat statistics is verified in this model. Our analytical results provide justification for the definition of quantum fluctuating heat via two-point measurements.

We also discuss the characteristic function of heat in the long-time limit or with the extremely weak/strong coupling strength. In the long-time limit, the form of the characteristic function of heat reflects complete thermalization of the system. In addition, from the analytical expressions of the heat statistics, we can immediately verify the exchange fluctuation theorem of heat. The phase-space formulation can be further utilized to study the joint statistics of work and heat in a driven open quantum system, which would be beneficial in exploring the fluctuations of power and efficiency in finite-time quantum heat engines. 

## Data Availability

Not applicable.

## Appendix A. Derivation to the Characteristic Function of Heat (17)

We show the detailed derivation to the characteristic function of heat $\chi_{\tau }\left(\nu \right)$ . First, we calculate the two Wigner functions ${\left[e^{i\nu H_{S}^{H}\left(t\right)}\right]}_{w}\left(z\right)$ and P(z) . Then, the final result Equation (17) is obtained from Equation (13).

### Appendix A.1. ${\left[e^{i\nu H_{S}^{H}\left(t\right)}\right]}_{w}\left(z\right)$

With the quadratic Hamiltonian $H_{S}^{H}\left(t\right)$ , the Wigner function ${\left[e^{i\nu H_{S}^{H}\left(t\right)}\right]}_{w}\left(z\right)$ is [78,81]

(A1)
$$\begin{array}{cc} {\left[e^{i\nu H_{S}^{H}\left(t\right)}\right]}_{w}\left(z\right) & =\frac{1}{\cos\left(\frac{\omega_{0}\hbar \nu }{2}\right)}\exp\left[i\frac{m_{0}\omega_{0}}{\hbar }\tan\left(\frac{\omega_{0}\hbar \nu }{2}\right)q_{0}^{2}\left(t\right)+i\frac{1}{m_{0}\hbar \omega_{0}}\tan\left(\frac{\omega_{0}\hbar \nu }{2}\right)p_{0}^{2}\left(t\right)\right] \end{array}$$

(A2)
$$\begin{array}{cc} \; & =\frac{1}{\cos\left(\frac{\omega_{0}\hbar \nu }{2}\right)}\exp\left[\frac{i}{2\hbar }z^{T}\left(t\right)\Lambda_{\nu z}z\left(t\right)\right], \end{array}$$

where z(t) gives the trajectory in the phase space determined by the initial point z(0)=z and $\Lambda_{\nu z}$ is a rank-2 diagonal matrix

(A3)
$$\Lambda_{\nu z}=\left(\begin{array}{cccc} 2m_{0}\omega_{0}\tan\left(\frac{\omega_{0}\hbar \nu }{2}\right)\\ \; & O\\ \; & \; & \frac{2}{m_{0}\omega_{0}}\tan\left(\frac{\omega_{0}\hbar \nu }{2}\right)\\ \; & \; & & O \end{array}\right),$$

with an N×N zero matrix O . The unlisted elements are zeros. The trajectory z(t) satisfies the classical equation of motion (also the equation of motion in the Heisenberg picture).

(A4)
$$\begin{array}{cc} {\dot{q}}_{0} & =\frac{p_{0}}{m_{0}}, \end{array}$$

(A5)
$$\begin{array}{cc} {\dot{q}}_{n} & =\frac{p_{n}}{m_{n}}, \end{array}$$

(A6)
$$\begin{array}{cc} {\dot{p}}_{0} & =-m_{0}{\tilde{\omega }}_{0}^{2}q_{0}+\sum_{n}C_{n}q_{n}, \end{array}$$

(A7)
$$\begin{array}{cc} {\dot{p}}_{n} & =-m_{n}\omega_{n}^{2}q_{n}+C_{n}q_{0}, \end{array}$$

with ${\tilde{\omega }}_{0}^{2}=\omega_{0}^{2}+\sum_{n=1}^{N}C_{n}^{2}/\left(m_{0}m_{n}\omega_{n}^{2}\right).$ The above differential equations can be rewritten into a compact form $\dot{z}\left(t\right)=Lz\left(t\right)$ . The trajectory z(t)=[q(t),p(t)] in the phase space characterizes the evolution of the composite system with the positions $q\left(t\right)=[q_{0}\left(t\right),...,q_{N}\left(t\right)]$ and the momenta $p\left(t\right)=[p_{0}\left(t\right),...,p_{N}\left(t\right)]$ and is related to the initial point by the dynamical map z(t)=exp(Lt)z(0). The (2N+2)×(2N+2) matrix L is explicitly

(A8)
$$L=\left(\begin{array}{cccccccccc} \; & \; & & \; & & \frac{1}{m_{0}}\\ \; & \; & & \; & & \; & \frac{1}{m_{1}}\\ \; & \; & & \; & & \; & & \frac{1}{m_{2}}\\ \; & \; & & \; & & \; & & \; & ...\\ \; & \; & & \; & & \; & & \; & & \frac{1}{m_{N}}\\ -m_{0}{\tilde{\omega }}_{0}^{2} & C_{1} & C_{2} & ... & C_{N}\\ C_{1} & -m_{1}\omega_{1}^{2}\\ C_{2} & \; & -m_{2}\omega_{2}^{2}\\ ... & \; & & ...\\ C_{N} & \; & & \; & -m_{N}\omega_{N}^{2} \end{array}\right),$$

and the matrix exponential is formally written as

(A9)
$$\exp\left(Lt\right)=\left(\begin{array}{cccccccc} \alpha_{0} & \alpha_{1} & ... & \alpha_{N} & \frac{\beta_{0}}{m_{0}} & \frac{\beta_{1}}{m_{1}} & ... & \frac{\beta_{N}}{m_{N}}\\ \gamma_{1} & \Lambda_{11} & ... & \Lambda_{1N} & \frac{\xi_{1}}{m_{0}} & \frac{\Delta_{11}}{m_{1}} & ... & \frac{\Delta_{1N}}{m_{N}}\\ ... & ... & ... & ... & ... & ... & ... & ...\\ \gamma_{N} & \Lambda_{N1} & ... & \Lambda_{NN} & \frac{\xi_{N}}{m_{0}} & \frac{\Delta_{N1}}{m_{1}} & ... & \frac{\Delta_{NN}}{m_{N}}\\ m_{0}{\dot{\alpha }}_{0} & m_{0}{\dot{\alpha }}_{1} & ... & m_{0}{\dot{\alpha }}_{N} & \frac{m_{0}}{m_{0}}{\dot{\beta }}_{0} & \frac{m_{0}}{m_{1}}{\dot{\beta }}_{1} & ... & \frac{m_{0}}{m_{N}}{\dot{\beta }}_{N}\\ m_{1}{\dot{\gamma }}_{1} & m_{1}{\dot{\Lambda }}_{11} & ... & m_{1}{\dot{\Lambda }}_{1N} & \frac{m_{1}}{m_{0}}{\dot{\xi }}_{1} & \frac{m_{1}}{m_{1}}{\dot{\Delta }}_{11} & ... & \frac{m_{1}}{m_{N}}{\dot{\Delta }}_{1N}\\ ... & ... & ... & ... & ... & ... & ... & ...\\ m_{N}{\dot{\gamma }}_{N} & m_{N}{\dot{\Lambda }}_{N1} & ... & m_{N}{\dot{\Lambda }}_{NN} & \frac{m_{N}}{m_{0}}{\dot{\xi }}_{N} & \frac{m_{N}}{m_{1}}{\dot{\Delta }}_{N1} & ... & \frac{m_{N}}{m_{N}}{\dot{\Delta }}_{NN} \end{array}\right).$$

We rewrite the quadratic form into $z^{T}\left(t\right)\Lambda_{\nu z}z\left(t\right)=z^{T}\left(0\right){\tilde{\Lambda }}_{\nu z}\left(t\right)z\left(0\right)$ with

(A10)
$$\begin{array}{cc} {\tilde{\Lambda }}_{\nu z}\left(t\right) & =\exp\left(L^{T}t\right)\Lambda_{\nu z}\exp\left(Lt\right). \end{array}$$

We next carry out every element in Equation (A9) through the Laplace transforms of Equations (A4)–(A7):

(A11)
$$\begin{array}{cc} s{\tilde{q}}_{0}\left(s\right)-q_{0}\left(0\right) & =\frac{{\tilde{p}}_{0}\left(s\right)}{m_{0}}, \end{array}$$

(A12)
$$\begin{array}{cc} s{\tilde{q}}_{n}\left(s\right)-q_{n}\left(0\right) & =\frac{{\tilde{p}}_{n}\left(s\right)}{m_{n}}, \end{array}$$

(A13)
$$\begin{array}{cc} s{\tilde{p}}_{0}\left(s\right)-p_{0}\left(0\right) & =-m_{0}{\tilde{\omega }}_{0}^{2}{\tilde{q}}_{0}\left(s\right)+\sum_{n}C_{n}{\tilde{q}}_{n}\left(s\right), \end{array}$$

(A14)
$$\begin{array}{cc} s{\tilde{p}}_{n}\left(s\right)-p_{n}\left(0\right) & =-m_{n}\omega_{n}^{2}{\tilde{q}}_{n}\left(s\right)+C_{n}{\tilde{q}}_{0}\left(s\right). \end{array}$$

Representing ${\tilde{q}}_{n}\left(s\right)$ and ${\tilde{p}}_{n}\left(s\right)$ with ${\tilde{q}}_{0}\left(s\right)$ and the initial conditions, we obtain

(A15)
$$\left\{s^{2}+{\tilde{\omega }}_{0}^{2}-\sum_{n}\left[\frac{C_{n}^{2}}{m_{0}m_{n}\left(s^{2}+\omega_{n}^{2}\right)}\right]\right\}{\tilde{q}}_{0}\left(s\right)={\dot{q}}_{0}\left(0\right)+sq_{0}\left(0\right)+\sum_{n}\frac{C_{n}}{m_{0}}\left[\frac{{\dot{q}}_{n}\left(0\right)+sq_{n}\left(0\right)}{s^{2}+\omega_{n}^{2}}\right].$$

Under the Ohmic spectral density Equation (4), the above equation is simplified to

(A16)
$$\left(s^{2}+\kappa s+\omega_{0}^{2}\right){\tilde{q}}_{0}\left(s\right)={\dot{q}}_{0}\left(0\right)+sq_{0}\left(0\right)+\sum_{n}\frac{C_{n}}{m_{0}}\left[\frac{{\dot{q}}_{n}\left(0\right)+sq_{n}\left(0\right)}{s^{2}+\omega_{n}^{2}}\right],$$

where the summation on the left-hand side of Equation (A15) can be approximately expressed as

(A17)
$$\begin{array}{cc} \sum_{n}\left[\frac{C_{n}^{2}}{m_{0}m_{n}\left(s^{2}+\omega_{n}^{2}\right)}\right] & \approx -\kappa s+\sum_{n}\frac{C_{n}^{2}}{m_{0}m_{n}\omega_{n}^{2}}, \end{array}$$

with a large cutoff frequency $\Omega_{0}$ . The inverse Laplace transform gives the differential equation of $q_{0}\left(t\right)$ as

(A18)
$${\ddot{q}}_{0}\left(t\right)+\kappa {\dot{q}}_{0}\left(t\right)+\omega_{0}^{2}q_{0}\left(t\right)=\underset{\mathrm{initial}\;\mathrm{velocity}\;\mathrm{change}}{\underset{︸}{-\kappa q_{0}\left(0\right)\delta \left(t\right)}}+\underset{\mathrm{stochastic}\;\mathrm{force}}{\underset{︸}{\sum_{n}\frac{C_{n}}{m_{0}}\left[{\dot{q}}_{n}\left(0\right)\frac{\sin(\omega_{n}t)}{\omega_{n}}+q_{n}\left(0\right)\cos\left(\omega_{n}t\right)\right]}}.$$

On the right-hand side, the second term presents the stochastic force induced by the heat bath; the first term indicates an abrupt velocity change $-\kappa q_{0}\left(0\right)$ of the system particle at the initial time t=0 [63,84,85]. The sudden change in velocity occurs for the system harmonic oscillator when the coupling between the system and the heat bath is switched on. Such an initial slippage is caused by the assumption of the initial product state. To avoid such an initial discontinuous problem, we drop the first term by considering the particle motion as starting at t=0+ [70]. Under such a modification, the Caldeira–Leggett model can reproduce the complete Langevin equation with an arbitrary friction coefficient κ for both the underdamped and the overdamped regimes and the heat distribution of the Caldeira–Leggett model approaches that of the classical Brownian motion described by the Kramers equation [86]. In Appendix A, for the classical counterpart of the Caldeira–Leggett model, we show that the initial slippage can be naturally eliminated by choosing another initial state.

After dropping the first term, Equation (A16) becomes

(A19)
$$\left(s^{2}+\kappa s+\omega_{0}^{2}\right){\tilde{q}}_{0}\left(s\right)={\dot{q}}_{0}\left(0\right)+\left(\kappa +s\right)q_{0}\left(0\right)+\sum_{n}\frac{C_{n}}{m_{0}}\left[\frac{{\dot{q}}_{n}\left(0\right)+sq_{n}\left(0\right)}{s^{2}+\omega_{n}^{2}}\right].$$

The solutions to ${\tilde{q}}_{0}\left(s\right)$ and ${\tilde{q}}_{n}\left(s\right)$ follow immediately as

(A20)
$$\begin{array}{cc} {\tilde{q}}_{0}\left(s\right) & =\frac{{\dot{q}}_{0}\left(0\right)+\left(\kappa +s\right)q_{0}\left(0\right)+\sum_{n}\frac{C_{n}}{m_{0}}\frac{{\dot{q}}_{n}\left(0\right)+sq_{n}\left(0\right)}{s^{2}+\omega_{n}^{2}}}{s^{2}+\kappa s+\omega_{0}^{2}}, \end{array}$$

(A21)
$$\begin{array}{cc} {\tilde{q}}_{n}\left(s\right) & =\frac{{\dot{q}}_{n}\left(0\right)+sq_{n}\left(0\right)}{s^{2}+\omega_{n}^{2}}+\frac{C_{n}}{m_{n}}\cdot \frac{{\dot{q}}_{0}\left(0\right)+\left(\kappa +s\right)q_{0}\left(0\right)+\sum_{l}\frac{C_{l}}{m_{0}}\frac{{\dot{q}}_{l}\left(0\right)+sq_{l}\left(0\right)}{s^{2}+\omega_{l}^{2}}}{\left(s^{2}+\omega_{n}^{2}\right)\left(s^{2}+\kappa s+\omega_{0}^{2}\right)}. \end{array}$$

With the inverse Laplace transform, the elements in the matrix $\exp\left(Lt\right)$ Equation (A9) are determined by

(A22)
$$\left(\begin{array}{c} q_{0}\left(t\right)\\ q_{1}\left(t\right)\\ ...\\ q_{N}\left(t\right) \end{array}\right)=\left(\begin{array}{cccccccccc} \alpha_{0} & \alpha_{1} & \alpha_{2} & ... & \alpha_{N} & \beta_{0} & \beta_{1} & \beta_{2} & ... & \beta_{N}\\ \gamma_{1} & \Lambda_{11} & \Lambda_{12} & ... & \Lambda_{1N} & \xi_{1} & \Delta_{11} & \Delta_{12} & ... & \Delta_{1N}\\ ... & ... & \; & ... & \; & ... & ... & \; & ...\\ \gamma_{N} & \Lambda_{N1} & \Lambda_{N2} & ... & \Lambda_{NN} & \xi_{N} & \Delta_{N1} & \Delta_{N2} & ... & \Delta_{NN} \end{array}\right)\left(\begin{array}{c} q_{0}\left(0\right)\\ q_{1}\left(0\right)\\ ...\\ q_{N}\left(0\right)\\ {\dot{q}}_{0}\left(0\right)\\ {\dot{q}}_{1}\left(0\right)\\ ...\\ {\dot{q}}_{N}\left(0\right) \end{array}\right),$$

where the elements in the matrix of the right-hand side are explicitly solved as [70]

(A23)
$$\begin{array}{cc} \alpha_{0} & =e^{-\frac{\kappa t}{2}}\left[\cos\left({\hat{\omega }}_{0}t\right)+\frac{\kappa }{2{\hat{\omega }}_{0}}\sin\left({\hat{\omega }}_{0}t\right)\right], \end{array}$$

(A24)
$$\begin{array}{cc} \beta_{0} & =\frac{e^{-\frac{\kappa t}{2}}}{{\hat{\omega }}_{0}}\sin\left({\hat{\omega }}_{0}t\right), \end{array}$$

(A25)
$$\begin{array}{cc} \alpha_{n} & =\frac{C_{n}}{m_{0}}f_{n}\left(t\right), \end{array}$$

(A26)
$$\begin{array}{cc} \beta_{n} & =\frac{C_{n}}{m_{0}}g_{n}\left(t\right), \end{array}$$

(A27)
$$\begin{array}{cc} \gamma_{n} & =\frac{C_{n}}{m_{n}}\left[f_{n}\left(t\right)+\kappa g_{n}\left(t\right)\right], \end{array}$$

(A28)
$$\begin{array}{cc} \xi_{n} & =\frac{C_{n}}{m_{n}}g_{n}\left(t\right), \end{array}$$

(A29)
$$\begin{array}{cc} \Lambda_{nl} & =\delta_{nl}\cos\left(\omega_{n}t\right)+\frac{C_{n}C_{l}}{m_{n}m_{0}}F_{nl}\left(t\right), \end{array}$$

(A30)
$$\begin{array}{cc} \Delta_{nl} & =\frac{\delta_{nl}}{\omega_{n}}\sin\left(\omega_{n}t\right)+\frac{C_{n}C_{l}}{m_{n}m_{0}}G_{nl}\left(t\right). \end{array}$$

The functions $f_{n}\left(t\right),\;g_{n}\left(t\right),\;F_{nl}\left(t\right)$ and $G_{nl}\left(t\right)$ are, explicitly,

(A31)
$$\begin{array}{cc} f_{n}\left(t\right) & =L^{-1}\left[\frac{s}{\left(s^{2}+\kappa s+\omega_{0}^{2}\right)\left(s^{2}+\omega_{n}^{2}\right)}\right], \end{array}$$

(A32)
$$\begin{array}{cc} g_{n}\left(t\right) & =L^{-1}\left[\frac{1}{\left(s^{2}+\kappa s+\omega_{0}^{2}\right)\left(s^{2}+\omega_{n}^{2}\right)}\right], \end{array}$$

(A33)
$$\begin{array}{cc} F_{nl}\left(t\right) & =L^{-1}\left[\frac{s}{\left(s^{2}+\kappa s+\omega_{0}^{2}\right)\left(s^{2}+\omega_{n}^{2}\right)\left(s^{2}+\omega_{l}^{2}\right)}\right], \end{array}$$

(A34)
$$\begin{array}{cc} G_{nl}\left(t\right) & =L^{-1}\left[\frac{1}{\left(s^{2}+\kappa s+\omega_{0}^{2}\right)\left(s^{2}+\omega_{n}^{2}\right)\left(s^{2}+\omega_{l}^{2}\right)}\right], \end{array}$$

where $L^{-1}\left(\cdot \right)$ denotes the inverse Laplace transform with $L\left(\cdot \right)=\int_{0}^{\infty }\left(\cdot \right)e^{-st}dt$ .

### Appendix A.2. P(z)

P(z) is the Wigner function of the state η(0) for the composite system [78,81]:

(A35)
$$P\left(z\right)=\frac{2\sinh\left(\frac{\beta^{\prime }\hbar \omega_{0}}{2}\right)}{\cosh\left[\frac{\left(\beta^{\prime }+i\nu \right)\hbar \omega_{0}}{2}\right]}\cdot \left[\prod_{n=1}^{N}2\tanh\left(\frac{\beta \hbar \omega_{n}}{2}\right)\right]\cdot \exp\left[-\frac{1}{2\hbar }z^{T}\Lambda_{\beta z}z\right],$$

where $\Lambda_{\beta z}$ is a (2N+2)×(2N+2) diagonal matrix

(A36)
$$\Lambda_{\beta z}=\mathrm{diag}\left(\lambda_{\beta^{\prime }q_{0}},\theta_{1},...,\theta_{N},\lambda_{\beta^{\prime }p_{0}},\mu_{1},...,\mu_{N}\right),$$

with the elements

(A37)
$$\begin{array}{cc} \theta_{n} & =2m_{n}\omega_{n}\tanh\left(\frac{\beta \hbar \omega_{n}}{2}\right), \end{array}$$

(A38)
$$\begin{array}{cc} \mu_{n} & =\frac{2}{m_{n}\omega_{n}}\tanh\left(\frac{\beta \hbar \omega_{n}}{2}\right), \end{array}$$

(A39)
$$\begin{array}{cc} \lambda_{\beta^{\prime }q_{0}} & =2m_{0}\omega_{0}\tanh\left[\frac{\left(\beta^{\prime }+i\nu \right)\hbar \omega_{0}}{2}\right], \end{array}$$

(A40)
$$\begin{array}{cc} \lambda_{\beta^{\prime }p_{0}} & =\frac{2}{m_{0}\omega_{0}}\tanh\left[\frac{\left(\beta^{\prime }+i\nu \right)\hbar \omega_{0}}{2}\right]. \end{array}$$

### Appendix A.3. Calculation of the Integral

With the explicit expressions of ${\left[e^{i\nu H_{S}^{H}\left(\tau \right)}\right]}_{w}\left(z\right)$ and P(z) , we perform the integral in Equation (13) and obtain the result of the characteristic function of heat

(A41)
$$\begin{array}{cc} \chi_{\tau }\left(\nu \right) & =\sqrt{\frac{\det\left(\Lambda_{\beta z}-i\Lambda_{\nu z}\right)}{\det\left[\Lambda_{\beta z}-i{\tilde{\Lambda }}_{\nu z}\left(\tau \right)\right]}}. \end{array}$$

We use the following integral formula:

(A42)
$$\int dxe^{-\frac{1}{2}x^{T}Tx}=\sqrt{\frac{{\left(2\pi \right)}^{\dim\left(T\right)}}{\det\left(T\right)}},$$

where all the eigenvalues of T have positive real parts.

By introducing a diagonal matrix $A=\Lambda_{\beta z}-i\Lambda_{\nu z}$ , we rewrite Equation (A41) as

(A43)
$$\begin{array}{cc} \chi_{\tau }\left(\nu \right) & =\sqrt{\frac{1}{\det\left(I+i\sqrt{A^{-1}}\left[\Lambda_{\nu z}-{\tilde{\Lambda }}_{\nu z}\left(\tau \right)\right]\sqrt{A^{-1}}\right)}}. \end{array}$$

Since ${\tilde{\Lambda }}_{\nu z}\left(\tau \right)$ is a rank-2 matrix, we rewrite it as

(A44)
$${\tilde{\Lambda }}_{\nu z}\left(\tau \right)=2m_{0}\omega_{0}\tan\left(\frac{\omega_{0}\hbar \nu }{2}\right)\left[v_{q_{0}}\left(\tau \right)v_{q_{0}}^{T}\left(\tau \right)+\frac{1}{m_{0}^{2}\omega_{0}^{2}}v_{p_{0}}\left(\tau \right)v_{p_{0}}^{T}\left(\tau \right)\right],$$

with the vectors

(A45)
$$\begin{array}{cc} v_{q_{0}}\left(\tau \right) & ={\left(\alpha_{0},\alpha_{1},...,\alpha_{N},\frac{\beta_{0}}{m_{0}},\frac{\beta_{1}}{m_{1}},...\frac{\beta_{N}}{m_{N}}\right)}^{T}, \end{array}$$

(A46)
$$\begin{array}{cc} v_{p_{0}}\left(\tau \right) & ={\left(m_{0}{\dot{\alpha }}_{0},m_{0}{\dot{\alpha }}_{1},...,m_{0}{\dot{\alpha }}_{N},{\dot{\beta }}_{0},\frac{m_{0}}{m_{1}}{\dot{\beta }}_{1},...,\frac{m_{0}}{m_{N}}{\dot{\beta }}_{N}\right)}^{T}. \end{array}$$

where the evolution time *t* in the terms $\alpha_{n}$ and $\beta_{n}$ is set to τ . We rewrite the matrix in the determinant (see Equation (A43)) as

(A47)
$$\sqrt{A^{-1}}\left(\Lambda_{\nu z}-{\tilde{\Lambda }}_{\nu z}\left(\tau \right)\right)\sqrt{A^{-1}}=MM^{T},$$

with the matrix

(A48)
$$M^{T}=\left(\begin{array}{c} \sqrt{2m_{0}\omega_{0}\tan\frac{\omega_{0}\hbar \nu }{2}}v_{q_{0}}^{T}\left(0\right)\\ \sqrt{\frac{2}{m_{0}\omega_{0}}\tan\frac{\omega_{0}\hbar \nu }{2}}v_{p_{0}}^{T}\left(0\right)\\ i\sqrt{2m_{0}\omega_{0}\tan\frac{\omega_{0}\hbar \nu }{2}}v_{q_{0}}^{T}\left(\tau \right)\\ i\sqrt{\frac{2}{m_{0}\omega_{0}}\tan\frac{\omega_{0}\hbar \nu }{2}}v_{p_{0}}^{T}\left(\tau \right) \end{array}\right)\sqrt{A^{-1}}.$$

The determinant in Equation (A43) can be simplified to

(A49)
$$\det\left(I+i\sqrt{A^{-1}}\left[\Lambda_{\nu z}-{\tilde{\Lambda }}_{\nu z}\left(\tau \right)\right]\sqrt{A^{-1}}\right)=\det\left(I_{4}+iM^{T}M\right),$$

where the right-hand side is the determinant of a 4×4 matrix and $I_{4}$ is the 4×4 identity matrix. Notice that the initial values of the two vectors are

(A50)
$$\begin{array}{cc} v_{q_{0}}\left(0\right) & ={\left(1,0,...0,0,0,...,0\right)}^{T}, \end{array}$$

(A51)
$$\begin{array}{cc} v_{p_{0}}\left(0\right) & ={\left(0,0,...0,1,0,...,0\right)}^{T}. \end{array}$$

The explicit result of $M^{T}M$ is obtained as

(A52)
$$M^{T}M=\Xi \left(\begin{array}{cccc} 1 & 0 & i\alpha_{0} & i{\dot{\alpha }}_{0}/\omega_{0}\\ 0 & 1 & i\omega_{0}\beta_{0} & i{\dot{\beta }}_{0}\\ i\alpha_{0} & i\omega_{0}\beta_{0} & -h_{11}\left(\tau \right) & -h_{12}\left(\tau \right)\\ i{\dot{\alpha }}_{0}/\omega_{0} & i{\dot{\beta }}_{0} & -h_{12}\left(\tau \right) & -h_{22}\left(\tau \right) \end{array}\right),$$

where the elements are functions of the final time τ and the functions $h_{11}\left(\tau \right)$ , $h_{12}\left(\tau \right)$ and $h_{22}\left(\tau \right)$ are

(A53)
$$\begin{array}{cc} h_{11}\left(\tau \right) & =\frac{2m_{0}\omega_{0}}{\Xi }\tan\left(\frac{\omega_{0}\hbar \nu }{2}\right)v_{q_{0}}^{T}\left(\tau \right)A^{-1}v_{q_{0}}\left(\tau \right), \end{array}$$

(A54)
$$\begin{array}{cc} h_{22}\left(\tau \right) & =\frac{2}{m_{0}\omega_{0}\Xi }\tan\left(\frac{\omega_{0}\hbar \nu }{2}\right)v_{p_{0}}^{T}\left(\tau \right)A^{-1}v_{p_{0}}\left(\tau \right), \end{array}$$

(A55)
$$\begin{array}{cc} h_{12}\left(\tau \right) & =\frac{2}{\Xi }\tan\left(\frac{\omega_{0}\hbar \nu }{2}\right)v_{q_{0}}^{T}\left(\tau \right)A^{-1}v_{p_{0}}\left(\tau \right), \end{array}$$

with

(A56)
$$\begin{array}{cc} v_{q_{0}}^{T}\left(t\right)A^{-1}v_{q_{0}}\left(t\right) & =\frac{\alpha_{0}^{2}+\omega_{0}^{2}\beta_{0}^{2}}{2m_{0}\omega_{0}\left\{\tanh\left[\frac{\left(\beta^{\prime }+i\nu \right)\hbar \omega_{0}}{2}\right]-i\tan\left(\frac{\omega_{0}\hbar \nu }{2}\right)\right\}}+\sum_{n=1}^{N}\left(\frac{\alpha_{n}^{2}}{\theta_{n}}+\frac{1}{\mu_{n}}\frac{\beta_{n}^{2}}{m_{n}^{2}}\right), \end{array}$$

(A57)
$$\begin{array}{cc} v_{p_{0}}^{T}\left(t\right)A^{-1}v_{p_{0}}\left(t\right) & =\frac{m_{0}\left({\dot{\alpha }}_{0}^{2}+\omega_{0}^{2}{\dot{\beta }}_{0}^{2}\right)}{2\omega_{0}\left\{\tanh\left[\frac{\left(\beta^{\prime }+i\nu \right)\hbar \omega_{0}}{2}\right]-i\tan\left(\frac{\omega_{0}\hbar \nu }{2}\right)\right\}}+m_{0}^{2}\sum_{n=1}^{N}\left(\frac{{\dot{\alpha }}_{n}^{2}}{\theta_{n}}+\frac{1}{\mu_{n}}\frac{{\dot{\beta }}_{n}^{2}}{m_{n}^{2}}\right), \end{array}$$

(A58)
$$\begin{array}{cc} v_{q_{0}}^{T}\left(t\right)A^{-1}v_{p_{0}}\left(t\right) & =\frac{\frac{d}{dt}\left(\alpha_{0}^{2}+\omega_{0}^{2}\beta_{0}^{2}\right)}{4\omega_{0}\left\{\tanh\left[\frac{\left(\beta^{\prime }+i\nu \right)\hbar \omega_{0}}{2}\right]-i\tan\left(\frac{\omega_{0}\hbar \nu }{2}\right)\right\}}+\frac{m_{0}}{2}\sum_{n=1}^{N}\frac{d}{dt}\left(\frac{\alpha_{n}^{2}}{\theta_{n}}+\frac{1}{\mu_{n}}\frac{\beta_{n}^{2}}{m_{n}^{2}}\right). \end{array}$$

The summations are replaced by the integral with the Ohmic spectral density and every element in Equation (A52) is carried out as

(A59)
$$\begin{array}{cc} \alpha_{0}\left(\tau \right) & =e^{-\frac{\kappa \tau }{2}}\left[\cos\left({\hat{\omega }}_{0}\tau \right)+\frac{\kappa \sin\left({\hat{\omega }}_{0}\tau \right)}{2{\hat{\omega }}_{0}}\right], \end{array}$$

(A60)
$$\begin{array}{cc} \beta_{0}\left(\tau \right) & =\frac{e^{-\frac{\kappa \tau }{2}}\sin\left({\hat{\omega }}_{0}\tau \right)}{{\hat{\omega }}_{0}}, \end{array}$$

(A61)
$$\begin{array}{cc} h_{11}\left(\tau \right) & =\Theta +e^{-\kappa \tau }\left[\frac{\omega_{0}^{2}}{{\hat{\omega }}_{0}^{2}}+\frac{\kappa \sin\left(2{\hat{\omega }}_{0}\tau \right)}{2{\hat{\omega }}_{0}}-\frac{\kappa^{2}\cos\left(2{\hat{\omega }}_{0}\tau \right)}{4{\hat{\omega }}_{0}^{2}}\right]\left(1-\Theta \right), \end{array}$$

(A62)
$$\begin{array}{cc} h_{22}\left(\tau \right) & =\Theta +e^{-\kappa \tau }\left[\frac{\omega_{0}^{2}}{{\hat{\omega }}_{0}^{2}}-\frac{\kappa \sin\left(2{\hat{\omega }}_{0}\tau \right)}{2{\hat{\omega }}_{0}}-\frac{\kappa^{2}\cos\left(2{\hat{\omega }}_{0}\tau \right)}{4{\hat{\omega }}_{0}^{2}}\right]\left(1-\Theta \right), \end{array}$$

(A63)
$$\begin{array}{cc} h_{12}\left(\tau \right) & =\frac{\kappa \omega_{0}e^{-\kappa \tau }}{2{\hat{\omega }}_{0}^{2}}\left(\Theta -1\right)\left[1-\cos\left(2{\hat{\omega }}_{0}\tau \right)\right]. \end{array}$$

Then, Equation (17) is obtained by directly calculating the determinant of a 4×4 matrix in Equation (A49).

## Appendix B. Classical Caldeira–Leggett Model

We consider the classical Caldeira–Leggett model, where coordinates and momenta commute with each other. To eliminate the initial slippage, the initial state is amended as a coupled state,

(A64)
$$\begin{array}{cc} \rho^{\mathrm{cl}}\left(z;0\right) & =\frac{e^{-\beta^{\prime }H_{S}\left(0\right)-\beta \left[H_{B}\left(0\right)+H_{SB}\left(0\right)\right]}}{Z^{\mathrm{cl}}\left(\beta^{\prime },\beta \right)}, \end{array}$$

which represents the probability density in the phase space of the composite system. The classical partition function is obtained by performing the integral in the phase space

(A65)
$$\begin{array}{cc} Z^{\mathrm{cl}}\left(\beta^{\prime },\beta \right) & =\;\int \int \;e^{-\beta^{\prime }H_{S}\left(0\right)-\beta \left[H_{B}\left(0\right)+H_{SB}\left(0\right)\right]}dq_{0}dq_{1}...dq_{N}dp_{0}dp_{1}...dp_{N} \end{array}$$

(A66)
$$\begin{array}{cc} \; & =\frac{2\pi }{\beta^{\prime }\omega_{0}}\prod_{n=1}^{N}\left(\frac{2\pi }{\beta \omega_{n}}\right), \end{array}$$

which is independent of the interaction (notice that the partition function of the quantum model relies on the interaction strength [68,87]).

We also define the classical fluctuating heat as the energy difference of the initial and the final system energy. For classical dynamics, the initial and the final states are directly represented by the points in the phase space and the measurements over the system can be applied without disturbing the composite system. Therefore, the characteristic function of heat is

(A67)
$$\chi_{\tau }^{\mathrm{cl}}\left(\nu \right)=\frac{\;\int \int \;e^{i\nu H_{S}\left(\tau \right)-\left(\beta^{\prime }+i\nu \right)H_{S}\left(0\right)-\beta \left[H_{B}\left(0\right)+H_{SB}\left(0\right)\right]}dq_{0}dq_{1}...dq_{N}dp_{0}dp_{1}...dp_{N}}{Z^{\mathrm{cl}}\left(\beta^{\prime },\beta \right)},$$

where the energy of the system $H_{S}\left(t\right)={\left[p_{0}\left(t\right)\right]}^{2}/\left(2m_{0}\right)+m_{0}\omega_{0}^{2}{\left[q_{0}\left(t\right)\right]}^{2}/2$ is determined by $p_{0}\left(t\right)$ and $q_{0}\left(t\right)$ associated with the initial point z . We choose a new set of initial variables $q_{0}$ , $q_{n}:=q_{n}-C_{n}q_{0}/\left(m_{n}\omega_{n}^{2}\right)$ , $p_{0}$ and $p_{n}$ in the following calculation.

We rewrite the evolution of the coordinate $q_{0}\left(t\right)$ of the system Equation (A16) as

(A68)
$$\left(s^{2}+\kappa s+\omega_{0}^{2}\right){\tilde{q}}_{0}\left(s\right)={\dot{q}}_{0}\left(0\right)+\left(s+\sum_{n}\frac{C_{n}^{2}}{m_{0}m_{n}\omega_{n}^{2}}\frac{s}{s^{2}+\omega_{n}^{2}}\right)q_{0}\left(0\right)+\sum_{n}\frac{C_{n}}{m_{0}}\frac{{\dot{q}}_{n}\left(0\right)+sq_{n}}{s^{2}+\omega_{n}^{2}}.$$

For the Ohmic spectral density, the summation in the second term is

(A69)
$$\sum_{n}\frac{C_{n}^{2}}{m_{0}m_{n}\omega_{n}^{2}}\frac{s}{s^{2}+\omega_{n}^{2}}=\kappa .$$

Thus, Equation (A68) naturally leads to Equation (A19) by substituting $q_{n}\left(0\right)$ into $q_{n}$ . The initial slippage is rationally eliminated by choosing a coupled initial state Equation (A64). In reality, the interaction between the system and the heat bath always exists and one cannot prepare the initial state of the composite system without the influence of the interaction. The initial state of the composite system is more likely in the coupled form Equation (A64). The heat bath encodes partial information of the system due to the interaction.

Similar to Equation (A10), the system energy at time *t* can be represented by the dynamical map as

(A70)
$$\begin{array}{cc} H_{S}\left(t\right) & =\frac{1}{2}z^{T}\left(t\right)\Lambda_{H_{S}}z\left(t\right) \end{array}$$

(A71)
$$\begin{array}{cc} \; & =\frac{1}{2}z^{T}\left(0\right){\tilde{\Lambda }}_{H_{S}}\left(t\right)z\left(0\right), \end{array}$$

with the following matrices [88]

(A72)
$$\begin{array}{cc} \Lambda_{H_{S}} & =\left(\begin{array}{cccc} m_{0}\omega_{0}^{2}\\ \; & O\\ \; & \; & \frac{1}{m_{0}}\\ \; & \; & & O \end{array}\right), \end{array}$$

and

(A73)
$${\tilde{\Lambda }}_{H_{S}}\left(t\right)=\exp\left(L^{T}t\right)\Lambda_{H_{S}}\exp\left(Lt\right).$$

The initial vector is now amended to

(A74)
$$z\left(0\right)={\left(q_{0}\left(0\right),q_{1},...,q_{n},p_{0}\left(0\right),...,p_{N}\left(0\right)\right)}^{T}.$$

The initial Hamiltonians $H_{S}\left(0\right)$ and $H_{B}\left(0\right)+H_{SB}\left(0\right)$ are

(A75)
$$\begin{array}{cc} H_{S}\left(0\right) & =\frac{1}{2}z^{T}\left(0\right)\Lambda_{H_{S}}z\left(0\right),\\ H_{B}\left(0\right)+H_{SB}\left(0\right) & =\sum_{n=1}^{N}\left(\frac{1}{2}\frac{p_{n}^{2}}{m_{n}}+\frac{1}{2}m_{n}\omega_{n}^{2}q_{n}^{2}\right) \end{array}$$

(A76)
$$\begin{array}{cc} \; & =\frac{1}{2}z^{T}\left(0\right)\Lambda_{H_{B}}z\left(0\right), \end{array}$$

with the matrix

(A77)
$$\Lambda_{H_{B}}=\mathrm{diag}\left(0,m_{1}\omega_{1}^{2},...,m_{N}\omega_{N}^{2},0,\frac{1}{m_{1}},...,\frac{1}{m_{N}}\right).$$

According to the integral Formula (A42), we carry out the characteristic function Equation (A67) into

(A78)
$$\chi_{\tau }^{\mathrm{cl}}\left(\nu \right)=\sqrt{\frac{\det\left[\beta^{\prime }\Lambda_{H_{S}}+\beta \Lambda_{H_{B}}\right]}{\det\left[\beta^{\prime }\Lambda_{H_{S}}+\beta \Lambda_{H_{B}}-i\nu \left({\tilde{\Lambda }}_{H_{S}}\left(\tau \right)-\Lambda_{H_{S}}\right)\right]}}.$$

For the classical limit ℏ→0 , we can verify

(A79)
$$\begin{array}{cc} \lim_{\hbar \to 0}\frac{\Lambda_{\beta z}}{\hbar } & =\left(\beta^{\prime }+i\nu \right)\Lambda_{H_{S}}+\beta \Lambda_{H_{B}}, \end{array}$$

(A80)
$$\begin{array}{cc} \lim_{\hbar \to 0}\frac{\Lambda_{\nu z}}{\hbar } & =\nu \Lambda_{H_{S}}, \end{array}$$

(A81)
$$\begin{array}{cc} \lim_{\hbar \to 0}\frac{{\tilde{\Lambda }}_{\nu z}\left(t\right)}{\hbar } & =\nu {\tilde{\Lambda }}_{H_{S}}\left(t\right), \end{array}$$

and obtain

(A82)
$$\lim_{\hbar \to 0}\chi_{\tau }\left(\nu \right)=\chi_{\tau }^{\mathrm{cl}}\left(\nu \right),$$

with $\chi_{\tau }\left(\nu \right)$ given in Equation (A41). The final result Equation (A78) is the same as Equation (25). Hence, we use the same notation.

## Appendix C. The Characteristic Function of Heat for the Classical Brownian Motion

We derive the characteristic function of heat for the classical Brownian motion. For an underdamped Brownian particle moving in a potential V(x) , the stochastic dynamics is described by the complete Langevin equation

(A83)
$$\ddot{x}+\kappa \dot{x}+\frac{1}{m}\frac{\partial V}{\partial x}=\frac{1}{m}F_{\mathrm{fluc}}\left(t\right).$$

The fluctuating force $F_{\mathrm{fluc}}\left(t\right)$ is a Gaussian white noise satisfying the fluctuation–dissipation relation

(A84)
$$\langle F_{\mathrm{fluc}}\left(t\right)F_{\mathrm{fluc}}\left(t^{\prime }\right)\rangle =2m\kappa k_{B}T\delta \left(t-t^{\prime }\right).$$

The evolution of the system state is characterized by the probability density function ρ(x,p;t) in the phase space. The stochastic dynamics is then described by the Kramers equation [86]

(A85)
$$\frac{\partial \rho }{\partial t}=L\left[\rho \right],$$

with the Liouville operator

(A86)
$$L\left[\rho \right]=-\frac{\partial }{\partial x}\left(\frac{p}{m}\rho \right)+\frac{\partial }{\partial p}\left[\kappa p\rho +\frac{\partial V(x)}{\partial x}\rho +\frac{\kappa m}{\beta }\frac{\partial \rho }{\partial p}\right].$$

Similarly, in the phase space, we calculate the characteristic function of heat for the classical Brownian motion

(A87)
$$\begin{array}{cc} \chi_{\tau }^{\mathrm{cl}}\left(\nu \right) & =\;\int \int \;dxdpe^{i\nu \left[\frac{p^{2}}{2m}+V\left(x\right)\right]}\eta \left(x,p;\tau \right), \end{array}$$

where a probability-density-like function η(x,p;t) also satisfies the dynamic Equation (A85) with the initial condition

(A88)
$$\eta \left(x,p;0\right)=e^{-i\nu \left[\frac{p^{2}}{2m}+V\left(x\right)\right]}\rho \left(x,p;0\right).$$

We consider the system potential as a harmonic potential $V\left(x\right)=m\omega_{0}^{2}x^{2}/2$ and the initial system state as an equilibrium state

(A89)
$$\rho \left(x,p;0\right)=\frac{1}{Z_{S}^{\mathrm{cl}}\left(\beta^{\prime }\right)}e^{-\beta^{\prime }\left(\frac{p^{2}}{2m}+\frac{1}{2}m\omega_{0}^{2}x^{2}\right)},$$

with the inverse temperature $\beta^{\prime }$ and the classical partition function $Z_{S}^{\mathrm{cl}}\left(\beta^{\prime }\right)=2\pi /\left(\beta^{\prime }\omega_{0}\right)$ . Under such conditions, the probability-density-like function η(x,p;t) is always in a quadratic form, assumed as

(A90)
$$\eta \left(x,p;t\right)=\frac{1}{Z_{S}^{\mathrm{cl}}\left(\beta^{\prime }\right)}e^{-\left[a\left(t\right)\frac{p^{2}}{2m}+b\left(t\right)\frac{1}{2}m\omega_{0}^{2}x^{2}+c\left(t\right)\omega_{0}xp+\Lambda \left(t\right)\right]}.$$

The Kramers Equation (A85) for η(x,p;t) leads to the following ordinary differential equations:

(A91)
$$\begin{array}{cc} \dot{\Lambda } & =-\kappa \left(1-\frac{a}{\beta }\right), \end{array}$$

(A92)
$$\begin{array}{cc} \dot{a} & =2\kappa a\left(1-\frac{a}{\beta }\right)-2\omega_{0}c, \end{array}$$

(A93)
$$\begin{array}{cc} \dot{b} & =2c\left(\omega_{0}-\frac{\kappa }{\beta }c\right), \end{array}$$

(A94)
$$\begin{array}{cc} \dot{c} & =\omega_{0}\left(a-b\right)+\kappa c-2\frac{\kappa }{\beta }ac, \end{array}$$

with the initial conditions $a\left(0\right)=b\left(0\right)=\beta^{\prime }+i\nu$ , c(0)=0 and Λ(0)=0 . According to the conservation of the probability ∫∫η(x,p;t)dxdp=const , the coefficient Λ(t) is obtained as

(A95)
$$\begin{array}{cc} e^{-\Lambda (t)} & =\frac{\sqrt{a\left(t\right)b\left(t\right)-c{\left(t\right)}^{2}}}{\beta^{\prime }+i\nu }. \end{array}$$

Substituting Equation (A90) into Equation (A87), we obtain the characteristic function for the classical Brownian motion as

(A96)
$$\chi_{\tau }^{\mathrm{cl}}\left(\nu \right)=\frac{\beta^{\prime }}{\beta^{\prime }+i\nu }\sqrt{\frac{a\left(\tau \right)b\left(\tau \right)-c{\left(\tau \right)}^{2}}{\left[a\left(\tau \right)-i\nu \right]\left[b\left(\tau \right)-i\nu \right]-c{\left(\tau \right)}^{2}}}.$$

To solve the nonlinear differential Equations (A92)–(A94), we introduce a new set of variables,

(A97)
$$\begin{array}{cc} A & =\frac{a}{ab-c^{2}}, \end{array}$$

(A98)
$$\begin{array}{cc} B & =\frac{b}{ab-c^{2}}, \end{array}$$

(A99)
$$\begin{array}{cc} C & =\frac{c}{ab-c^{2}}, \end{array}$$

and obtain the linear differential equations

(A100)
$$\begin{array}{cc} \frac{dA}{dt} & =-2\omega_{0}C, \end{array}$$

(A101)
$$\begin{array}{cc} \frac{dB}{dt} & =2\omega_{0}C-2\kappa B+2\frac{\kappa }{\beta }, \end{array}$$

(A102)
$$\begin{array}{cc} \frac{dC}{dt} & =\omega_{0}\left(A-B\right)-\kappa C, \end{array}$$

with the initial conditions $A\left(0\right)=B\left(0\right)=1/\left(\beta^{\prime }+i\nu \right)$ and C(0)=0 . The characteristic function Equation (A96) becomes

(A103)
$$\chi_{\tau }^{\mathrm{cl}}\left(\nu \right)=\frac{\beta^{\prime }}{\beta^{\prime }+i\nu }\sqrt{\frac{1}{1-i\nu \left(A+B\right)-\nu^{2}\left(AB-C^{2}\right)}}.$$

The solutions to Equations (A100)–(A102) are

(A104)
$$\begin{array}{cc} A(t) & =-\frac{e^{-\kappa t}\left[\kappa^{2}\cos\left(2{\hat{\omega }}_{0}t\right)-2{\hat{\omega }}_{0}\kappa \sin\left(2{\hat{\omega }}_{0}t\right)-4\omega_{0}^{2}\right]}{4\beta {\hat{\omega }}_{0}^{2}}\frac{\beta -\beta^{\prime }-i\nu }{\beta^{\prime }+i\nu }+\frac{1}{\beta }, \end{array}$$

(A105)
$$\begin{array}{cc} B(t) & =-\frac{e^{-\kappa t}\left[\kappa^{2}\cos\left(2{\hat{\omega }}_{0}t\right)+2{\hat{\omega }}_{0}\kappa \sin\left(2{\hat{\omega }}_{0}t\right)-4\omega_{0}^{2}\right]}{4\beta {\hat{\omega }}_{0}^{2}}\frac{\beta -\beta^{\prime }-i\nu }{\beta^{\prime }+i\nu }+\frac{1}{\beta }, \end{array}$$

(A106)
$$\begin{array}{cc} C(t) & =-\frac{2e^{-\kappa t}\kappa \omega_{0}\left[\cos\left(2{\hat{\omega }}_{0}t\right)-1\right]}{4\beta {\hat{\omega }}_{0}^{2}}\frac{\beta -\beta^{\prime }-i\nu }{\beta^{\prime }+i\nu }, \end{array}$$

with ${\hat{\omega }}_{0}=\sqrt{\omega_{0}^{2}-\kappa^{2}/4}$ . Plugging the solutions into Equation (A103), we immediately obtain Equation (25). We remark that the heat distribution of the classical Brownian motion was obtained by the path-integral method in Ref. [58], but they only consider the initial temperature of the system to be the same as that of the bath.

### Appendix C.1. The Long-Time Limit

After sufficiently long relaxation time, the solutions a(t) , b(t) and c(t) to Equations (A92)–(A94) eventually approach a(∞)=b(∞)=β and c(∞)=0 . The long-time limit of Equation (A96) reproduces Equation (33).

### Appendix C.2. The Underdamped Limit

In the underdamped limit $\kappa /\omega_{0}\to 0$ , the differential Equations (A92)–(A94) are reduced to

(A107)
$$\dot{a}=\kappa a\left(1-\frac{a}{\beta }\right),$$

with b=a and c=0 . The solution is

(A108)
$$a\left(t\right)=\frac{\beta (\beta^{\prime }+i\nu )}{\beta^{\prime }+i\nu +\left(\beta -\beta^{\prime }-i\nu \right)e^{-\kappa t}},$$

and Equation (A96) becomes

(A109)
$$\chi_{\tau }^{w,\mathrm{cl}}\left(\nu \right)=\frac{\beta \beta^{\prime }}{\left(\beta -i\nu \right)\left(\beta^{\prime }+i\nu \right)\left(1-e^{-\kappa \tau }\right)+\beta \beta^{\prime }e^{-\kappa \tau }}.$$

It can be checked that Equation (37) reproduces Equation (A109) in the classical limit ℏ→0 .

### Appendix C.3. The Overdamped Limit

In the overdamped limit $\kappa /\omega_{0}\to \infty$ , the differential Equations (A92)–(A94) are reduced to

(A110)
$$\begin{array}{cc} \dot{a} & =2\kappa a\left(1-\frac{a}{\beta }\right), \end{array}$$

(A111)
$$\begin{array}{cc} \dot{b} & =2\omega_{0}c\left(1-\frac{\kappa }{\beta \omega_{0}}c\right), \end{array}$$

(A112)
$$\begin{array}{cc} 0 & =\left(\kappa c+\omega_{0}a\right)-2\frac{\kappa }{\beta }ac-b\omega_{0}. \end{array}$$

Eliminating *c* in Equation (A112), Equation (A111) becomes

(A113)
$$\dot{b}=\frac{2\omega_{0}^{2}}{\kappa }\frac{\left(\beta -a-b\right)\left(b-a\right)}{\left(\beta -2a\right)\left(1-2\frac{a}{\beta }\right)}.$$

Notice that, in the overdamped limit, the relaxation timescales of the momentum and the coordinate are separated. We can substitute a=β in Equation (A113) and obtain

(A114)
$$\dot{b}=\frac{2\omega_{0}^{2}}{\kappa }b\left(1-\frac{b}{\beta }\right).$$

With the initial condition $a\left(0\right)=\beta^{\prime }+i\nu$ and $b\left(0\right)=\beta^{\prime }+i\nu$ , the solutions are

(A115)
$$\begin{array}{cc} a(t) & =\frac{\left(\beta^{\prime }+i\nu \right)\beta }{\beta^{\prime }+i\nu +\left(\beta -\beta^{\prime }-i\nu \right)e^{-2\kappa t}}, \end{array}$$

(A116)
$$\begin{array}{cc} b(t) & =\frac{\left(\beta^{\prime }+i\nu \right)\beta }{\beta^{\prime }+i\nu +\left(\beta -\beta^{\prime }-i\nu \right)e^{-\frac{2\omega_{0}^{2}}{\kappa }t}}. \end{array}$$

We substitute Equations (A115), (A116) and c(t)≈0 into Equation (A96) and obtain

(A117)
$$\begin{array}{cc} \chi_{\tau }^{s,\mathrm{cl}}\left(\nu \right)= & \frac{\beta \beta^{\prime }}{\sqrt{\left(\beta -i\nu \right)\left(\beta^{\prime }+i\nu \right)\left(1-e^{-2\kappa \tau }\right)+\beta \beta^{\prime }e^{-2\kappa \tau }}}\\ \; & \times \frac{1}{\sqrt{\left(\beta -i\nu \right)\left(\beta^{\prime }+i\nu \right)\left(1-e^{-\frac{2\omega_{0}^{2}}{\kappa }\tau }\right)+\beta \beta^{\prime }e^{-\frac{2\omega_{0}^{2}}{\kappa }\tau }}}. \end{array}$$

It can be checked that Equation (38) reproduces Equation (A117) in the classical limit ℏ→0 .

## Appendix D. The Characteristic Function of Heat for Complete Thermalization

We derive the characteristic function of heat for a complete thermalization process, Equation (34), in the main content. For a complete thermalization process (typically with infinite relaxation time), the information of the initial state is completely forgotten and the final state is always an equilibrium state at the inverse temperature β of the heat bath,

(A118)
$$\gamma_{\mathrm{th},l^{\prime }l}=p_{l^{\prime }}^{\mathrm{eq}},$$

regardless of the initial state *l*. Therefore, the characteristic function of heat for complete thermalization is $\chi_{\mathrm{th}}\left(\nu \right)=\sum_{l^{\prime },l}\exp\left[i\nu \left(E_{l^{\prime }}^{S}-E_{l}^{S}\right)\right]p_{l}p_{l^{\prime }}^{\mathrm{eq}}$ . We immediately obtain Equation (34) by plugging into the initial distribution $p_{l}=\exp\left(-\beta^{\prime }E_{l}^{S}\right)/Z_{S}\left(\beta^{\prime }\right)$ and the final distribution $p_{l^{\prime }}^{\mathrm{eq}}=\exp\left(-\beta E_{l^{\prime }}^{S}\right)/Z_{S}\left(\beta \right)$ , where $Z_{S}\left(\beta^{\prime }\right)=\sum_{l}\exp\left(-\beta^{\prime }E_{l}^{S}\right)$ is the partition function of the system at the inverse temperature $\beta^{\prime }$ .

References and Notes

## References

[1] Gallavotti G., Cohen E.G.D. (1995). Dynamical Ensembles in Nonequilibrium Statistical Mechanics. Phys. Rev. Lett..

[2] Jarzynski C. (1997). Nonequilibrium Equality for Free Energy Differences. Phys. Rev. Lett..

[3] Crooks G.E. (1999). Entropy production fluctuation theorem and the nonequilibrium work relation for free energy differences. Phys. Rev. E.

[4] Jarzynski C., Wójcik D.K. (2004). Classical and Quantum Fluctuation Theorems for Heat Exchange. Phys. Rev. Lett..

[5] Jarzynski C. (2011). Equalities and Inequalities: Irreversibility and the Second Law of Thermodynamics at the Nanoscale. Annu. Rev. Condens. Matter Phys..

[6] Sekimoto K. (2010). Stochastic Energetics.

[7] Seifert U. (2012). Stochastic thermodynamics, fluctuation theorems and molecular machines. Rep. Prog. Phys..

[8] Seifert U. (2005). Entropy Production along a Stochastic Trajectory and an Integral Fluctuation Theorem. Phys. Rev. Lett..

[9] Esposito M., Harbola U., Mukamel S. (2009). Nonequilibrium fluctuations, fluctuation theorems, and counting statistics in quantum systems. Rev. Mod. Phys..

[10] Campisi M., Hänggi P., Talkner P. (2011). Colloquium: Quantum fluctuation relations: Foundations and applications. Rev. Mod. Phys..

[11] Klages R. (2013). Nonequilibrium Statistical Physics of Small Systems: Fluctuation Relations and Beyond.

[12] Horodecki M., Oppenheim J. (2013). Fundamental limitations for quantum and nanoscale thermodynamics. Nat Commun.

[13] Ciliberto S. (2017). Experiments in Stochastic Thermodynamics: Short History and Perspectives. Phys. Rev. X.

[14] Tasaki H. (2000). Jarzynski Relations for Quantum Systems and Some Applications. arXiv.

[15] Kurchan J. (2000). A Quantum Fluctuation Theorem. arXiv.

[16] Talkner P., Lutz E., Hänggi P. (2007). Fluctuation theorems: Work is not an observable. Phys. Rev. E.

[17] Deffner S., Lutz E. (2008). Nonequilibrium work distribution of a quantum harmonic oscillator. Phys. Rev. E.

[18] Liu F. (2014). Calculating work in adiabatic two-level quantum Markovian master equations: A characteristic function method. Phys. Rev. E.

[19] Zhu L., Gong Z., Wu B., Quan H.T. (2016). Quantum-classical correspondence principle for work distributions in a chaotic system. Phys. Rev. E.

[20] Funo K., Quan H. (2018). Path Integral Approach to Quantum Thermodynamics. Phys. Rev. Lett..

[21] Salazar D.S.P., Lira S.A. (2019). Stochastic thermodynamics of nonharmonic oscillators in high vacuum. Phys. Rev. E.

[22] Jarzynski C., Quan H., Rahav S. (2015). Quantum-Classical Correspondence Principle for Work Distributions. Phys. Rev. X.

[23] Deffner S., Paz J.P., Zurek W.H. (2016). Quantum work and the thermodynamic cost of quantum measurements. Phys. Rev. E.

[24] García-Mata I., Roncaglia A.J., Wisniacki D.A. (2017). Quantum-to-classical transition in the work distribution for chaotic systems. Phys. Rev. E.

[25] Fei Z., Quan H. (2020). Nonequilibrium Green’s Function’s Approach to the Calculation of Work Statistics. Phys. Rev. Lett..

[26] Qiu T., Fei Z., Pan R., Quan H.T. (2020). Path-integral approach to the calculation of the characteristic function of work. Phys. Rev. E.

[27] Zurek W.H. (1991). Decoherence and the Transition from Quantum to Classical. Phys. Today.

[28] Zurek W.H. (2003). Decoherence, einselection, and the quantum origins of the classical. Rev. Mod. Phys..

[29] Saito K., Dhar A. (2007). Fluctuation Theorem in Quantum Heat Conduction. Phys. Rev. Lett..

[30] Dubi Y., Ventra M.D. (2011). Colloquium: Heat flow and thermoelectricity in atomic and molecular junctions. Rev. Mod. Phys..

[31] Thingna J., García-Palacios J.L., Wang J.S. (2012). Steady-state thermal transport in anharmonic systems: Application to molecular junctions. Phys. Rev. B.

[32] Wang J.S., Agarwalla B.K., Li H., Thingna J. (2014). Nonequilibrium Green’s function method for quantum thermal transport. Front. Phys..

[33] Thingna J., Manzano D., Cao J. (2016). Dynamical signatures of molecular symmetries in nonequilibrium quantum transport. Sci. Rep..

[34] He D., Thingna J., Wang J.S., Li B. (2016). Quantum thermal transport through anharmonic systems: A self-consistent approach. Phys. Rev. B.

[35] Segal D., Agarwalla B.K. (2016). Vibrational Heat Transport in Molecular Junctions. Ann. Phys. Chem..

[36] Kilgour M., Agarwalla B.K., Segal D. (2019). Path-integral methodology and simulations of quantum thermal transport: Full counting statistics approach. J. Chem. Phys..

[37] Wang C., Ren J., Cao J. (2017). Unifying quantum heat transfer in a nonequilibrium spin-boson model with full counting statistics. Phys. Rev. A.

[38] Aurell E., Donvil B., Mallick K. (2020). Large deviations and fluctuation theorem for the quantum heat current in the spin-boson model. Phys. Rev. E.

[39] Denzler T., Lutz E. (2018). Heat distribution of a quantum harmonic oscillator. Phys. Rev. E.

[40] Salazar D.S.P., Macêdo A.M.S., Vasconcelos G.L. (2019). Quantum heat distribution in thermal relaxation processes. Phys. Rev. E.

[41] Popovic M., Mitchison M.T., Strathearn A., Lovett B.W., Goold J., Eastham P.R. (2021). Quantum Heat Statistics with Time-Evolving Matrix Product Operators. PRX Quantum.

[42] Karsten Balzer M.B. (2012). Nonequilibrium Green’s Functions Approach to Inhomogeneous Systems.

[43] Esposito M., Ochoa M.A., Galperin M. (2015). Quantum Thermodynamics: A Nonequilibrium Green’s Function Approach. Phys. Rev. Lett..

[44] Polanco C.A. (2021). Nonequilibrium Green’s functions (NEGF) in vibrational energy transport: A topical review. Nanoscale Microscale Thermophys. Eng..

[45] Aron C., Biroli G., Cugliandolo L.F. (2010). Symmetries of generating functionals of Langevin processes with colored multiplicative noise. J. Stat. Mech..

[46] Mallick K., Moshe M., Orland H. (2011). A field-theoretic approach to non-equilibrium work identities. J. Phys. A.

[47] Carrega M., Solinas P., Braggio A., Sassetti M., Weiss U. (2015). Functional integral approach to time-dependent heat exchange in open quantum systems: General method and applications. New J. Phys..

[48] Funo K., Quan H.T. (2018). Path integral approach to heat in quantum thermodynamics. Phys. Rev. E.

[49] Yeo J. (2019). Symmetry and its breaking in a path-integral approach to quantum Brownian motion. Phys. Rev. E.

[50] Fogedby H.C. (2020). Heat fluctuations in equilibrium. J. Stat. Mech. Theory Exp..

[51] Van Zon R., Cohen E.G.D. (2004). Extended heat-fluctuation theorems for a system with deterministic and stochastic forces. Phys. Rev. E.

[52] Imparato A., Peliti L., Pesce G., Rusciano G., Sasso A. (2007). Work and heat probability distribution of an optically driven Brownian particle: Theory and experiments. Phys. Rev. E.

[53] Fogedby H.C., Imparato A. (2009). Heat distribution function for motion in a general potential at low temperature. J. Phys. A Math. Theor..

[54] Chatterjee D., Cherayil B.J. (2010). Exact path-integral evaluation of the heat distribution function of a trapped Brownian oscillator. Phys. Rev. E.

[55] Gomez-Solano J.R., Petrosyan A., Ciliberto S. (2011). Heat Fluctuations in a Nonequilibrium Bath. Phys. Rev. Lett..

[56] Salazar D.S.P., Lira S.A. (2016). Exactly solvable nonequilibrium Langevin relaxation of a trapped nanoparticle. J. Phys. A Math. Theor..

[57] Pagare A., Cherayil B.J. (2019). Stochastic thermodynamics of a harmonically trapped colloid in linear mixed flow. Phys. Rev. E.

[58] Paraguassú P.V., Aquino R., Morgado W.A.M. (2021). The Heat Distribution of the Underdamped Langevin Equation. arXiv.

[59] Gupta D., Sivak D.A. (2021). Heat fluctuations in a harmonic chain of active particles. Phys. Rev. E.

[60] Esposito M., Ochoa M.A., Galperin M. (2015). Nature of heat in strongly coupled open quantum systems. Phys. Rev. B.

[61] Talkner P., Hänggi P. (2016). Open system trajectories specify fluctuating work but not heat. Phys. Rev. E.

[62] Talkner P., Hänggi P. (2020). Colloquium: Statistical mechanics and thermodynamics at strong coupling: Quantum and classical. Rev. Mod. Phys..

[63] Bez W. (1980). Microscopic preparation and macroscopic motion of a Brownian particle. Z. Phys. B.

[64] Caldeira A., Leggett A. (1983). Path integral approach to quantum Brownian motion. Phys. A.

[65] Caldeira A., Leggett A. (1983). Quantum tunnelling in a dissipative system. Ann. Phys..

[66] Unruh W.G., Zurek W.H. (1989). Reduction of a wave packet in quantum Brownian motion. Phys. Rev. D.

[67] Breuer H.P., Petruccione F. (2007). The Theory of Open Quantum Systems.

[68] Weiss U. (2008). Quantum Dissipative Systems.

[B69-entropy-23-01602] 69.Usually the quantum fluctuating heat is defined via two-point measurements over the heat bath. When the Hamiltonian of the system is time-independent, the internal energy change of the system is completely caused by the heat exchange. The quantum fluctuating heat can thus be alternatively defined via two-point measurements over the system, whose number of degrees of freedom is much smaller than that of the heat bath. Hence, the calculation of the heat statistics can be significantly simplified under this definition

[70] Yu L.H., Sun C.P. (1994). Evolution of the wave function in a dissipative system. Phys. Rev. A.

[71] Wigner E. (1932). On the Quantum Correction For Thermodynamic Equilibrium. Phys. Rev..

[72] Hillery M., O’Connell R., Scully M., Wigner E. (1984). Distribution functions in physics: Fundamentals. Phys. Rep..

[73] Polkovnikov A. (2010). Phase space representation of quantum dynamics. Ann. Phys..

[74] Fei Z., Quan H.T., Liu F. (2018). Quantum corrections of work statistics in closed quantum systems. Phys. Rev. E.

[75] Qian Y., Liu F. (2019). Computing characteristic functions of quantum work in phase space. Phys. Rev. E.

[76] Brodier O., Mallick K., de Almeida A.M.O. (2020). Semiclassical work and quantum work identities in Weyl representation. J. Phys. A Math. Theor..

[77] Qiu T., Fei Z., Pan R., Quan H.T. (2020). Quantum corrections to the entropy and its application in the study of quantum Carnot engines. Phys. Rev. E.

[78] Qiu T., Quan H.T. (2021). Quantum corrections to the entropy in a driven quantum Brownian motion model. Commun. Theor. Phys..

[79] Hu B.L., Paz J.P., Zhang Y. (1992). Quantum Brownian motion in a general environment: Exact master equation with nonlocal dissipation and colored noise. Phys. Rev. D.

[80] Karrlein R., Grabert H. (1997). Exact time evolution and master equations for the damped harmonic oscillator. Phys. Rev. E.

[81] Ford G.W., O’Connell R.F. (2001). Exact solution of the Hu-Paz-Zhang master equation. Phys. Rev. D.

[82] Salazar D.S.P. (2020). Work distribution in thermal processes. Phys. Rev. E.

[83] Chen Y.H., Chen J.F., Fei Z., Quan H.T. (2021). A microscopic theory of Curzon-Ahlborn heat engine. arXiv.

[84] Cañizares J.S., Sols F. (1994). Translational symmetry and microscopic preparation in oscillator models of quantum dissipation. Phys. A.

[85] Ju K.K., Guo C.X., Pan X.Y. (2017). Initial-Slip Term Effects on the Dissipation-Induced Transition of a Simple Harmonic Oscillator. Chin. Phys. Lett..

[86] Kramers H.A. (1940). Brownian motion in a field of force and the diffusion model of chemical reactions. Physica.

[87] Grabert H., Weiss U., Talkner P. (1984). Quantum theory of the damped harmonic oscillator. Z. Phys. B.

[B88-entropy-23-01602] 88.Strictly, the substitution requires to amend γn, Λnm, ξn, Δnm accordingly, but we only require q0(t) and p0(t) to calculate the characteristic function of heat, so we skip the further amendment.

